# Repetitive mild traumatic brain injury in mice triggers a slowly developing cascade of long-term and persistent behavioral deficits and pathological changes

**DOI:** 10.1186/s40478-021-01161-2

**Published:** 2021-04-06

**Authors:** Xiaoyun Xu, Matthew Cowan, Flavio Beraldo, Amy Schranz, Patrick McCunn, Nicole Geremia, Zalman Brown, Maitray Patel, Karen L. Nygard, Reza Khazaee, Lihong Lu, Xingyu Liu, Michael J. Strong, Gregory A. Dekaban, Ravi Menon, Robert Bartha, Mark Daley, Haojie Mao, Vania Prado, Marco A. M. Prado, Lisa Saksida, Tim Bussey, Arthur Brown

**Affiliations:** 1 Translational Neuroscience Group, Robarts Research Institute, Schulich School of Medicine and Dentistry, Western University, 1151 Richmond Street North, London, ON N6A 5B7 Canada; 2 Department of Medical Biophysics, Schulich School of Medicine and Dentistry, The University of Western Ontario, 1151 Richmond Street North, London, ON N6A 5B7 Canada; 3 Department of Computer Science, Schulich School of Medicine and Dentistry, The University of Western Ontario, 1151 Richmond Street North, London, ON N6A 5B7 Canada; 4 The Biotron Experimental Climate Change Research Centre, The University of Western Ontario, 1151 Richmond Street North, London, ON N6A 5B7 Canada; 5 Department of Mechanical and Materials Engineering, Schulich School of Medicine and Dentistry, The University of Western Ontario, 1151 Richmond Street North, London, ON N6A 5B7 Canada; 6 Department of Clinical Neurological Sciences, Schulich School of Medicine and Dentistry, The University of Western Ontario, London, ON N6A 5B7 Canada; 7 Molecular Medicine Research Laboratories, Robarts Research Institute, Schulich School of Medicine and Dentistry, Western University, London, ON Canada; 8 Department of Microbiology and Immunology, Schulich School of Medicine and Dentistry, The University of Western Ontario, 1151 Richmond Street North, London, ON N6A 5B7 Canada; 9 Centre for Functional and Metabolic Mapping, Robarts Research Institute, The University of Western Ontario, 1151 Richmond Street North, London, ON N6A 5B7 Canada; 10 Department of Physiology and Pharmacology, Schulich School of Medicine and Dentistry, The University of Western Ontario, 1151 Richmond Street North, London, ON N6A 5B7 Canada; 11 The Vector Institute for Artificial Intelligence, 661 University Ave Suite 710, Toronto, Canada; 12 Department of Anatomy and Cell Biology, The University of Western Ontario, 1151 Richmond Street North, London, ON N6A 5B7 Canada

**Keywords:** Concussion, 5-Choice serial reaction time test, White matter pathology, Tauopathy, Experimental brain injury, Traumatic encephalopathy, Metabolomics, Magnetic resonance imaging

## Abstract

We have previously reported long-term changes in the brains of non-concussed varsity rugby players using magnetic resonance spectroscopy (MRS), diffusion tensor imaging (DTI) and functional magnetic imaging (fMRI). Others have reported cognitive deficits in contact sport athletes that have not met the diagnostic criteria for concussion. These results suggest that repetitive mild traumatic brain injuries (rmTBIs) that are not severe enough to meet the diagnostic threshold for concussion, produce long-term consequences. We sought to characterize the neuroimaging, cognitive, pathological and metabolomic changes in a mouse model of rmTBI. Using a closed-skull model of mTBI that when scaled to human leads to rotational and linear accelerations far below what has been reported for sports concussion athletes, we found that 5 daily mTBIs triggered two temporally distinct types of pathological changes. First, during the first days and weeks after injury, the rmTBI produced diffuse axonal injury, a transient inflammatory response and changes in diffusion tensor imaging (DTI) that resolved with time. Second, the rmTBI led to pathological changes that were evident months after the injury including: changes in magnetic resonance spectroscopy (MRS), altered levels of synaptic proteins, behavioural deficits in attention and spatial memory, accumulations of pathologically phosphorylated tau, altered blood metabolomic profiles and white matter ultrastructural abnormalities. These results indicate that exceedingly mild rmTBI, in mice, triggers processes with pathological consequences observable months after the initial injury.

**Supplementary Information:**

The online version contains supplementary material available at 10.1186/s40478-021-01161-2.

## Introduction

Traumatic brain injury (TBI) was defined in a 2010 position statement as an alteration in brain function, or other evidence of brain pathology caused by an external force [70]. Worldwide estimates suggest that there are 50–60 million new TBIs each year [61] with approximately 3.5 million in the United States alone [20]. TBI severity can be classified as either mild, moderate or severe by applying the Glasgow Coma Scale (GCS) within 24 h of trauma [48]. A GCS score of 13–15 is considered a mild TBI (mTBI) while a GCS score of 9–12 is considered moderate and a GCS score of 8 or less is considered severe TBI. The overwhelming majority (75–90%) of TBIs are mild [27, 47, 61] with the acknowledgment that the incidence of mTBI is likely grossly underestimated because many patients with mTBI do not seek medical treatment [76]. Thus, understanding the pathophysiology of mTBI is a critical medical issue. The importance of repetitive mTBIs (rmTBIs) is underscored by the finding that a history of rmTBIs is associated with an increased risk of developing chronic traumatic encephalopathy (CTE) [68, 69, 102] and of dementia [4, 28, 72, 81]. The association of a history of mTBI with the later development of dementia and the recognition that many patients with mTBI have symptoms that persist for months or years [3] raises the question: just how mild are mTBIs?

Although we recognize that the term concussion is problematic [48, 94] we will use it to denote a constellation of symptoms that may or may not be transient (headache, nausea, dizziness, etc.) after a mTBI. There is increasing evidence that patients may experience a mTBI in the absence of symptoms or signs that would lead to a diagnosis of concussion. In a study of women’s varsity rugby, we demonstrated that non-concussed rugby athletes have elevated levels of glutamine and of glutamine/creatine ratios by magnetic resonance spectroscopy (MRS) during in-season play compared to off-season [93]. In a fMRI and DTI study comparing concussion-free and concussed women varsity rugby players, we demonstrated white matter and functional connectivity changes in asymptomatic contact sport athletes [63]. We have also reported differences in DTI, functional connectivity and in MRS in concussion-free athletes playing contact sports compared to concussion-free athletes involved in non-contact sports (swimmers and rowers) [62, 92]. These results are consistent with other fMRI studies that likewise show changes in functional connectivity in contact sport athletes that have not been diagnosed with concussion [100]. Studies have also shown cognitive impairment in athletes playing contact sports that are reported to be concussion-free [50, 66, 104]. Finally, cognitive and imaging studies of non-concussed athletes playing contact sports demonstrate a correlation between the number of years of play and abnormalities observed [2, 104]. These studies, when considered together, suggest that significant changes in brain metabolism, white matter integrity, functional connectivity and cognitive function may occur after rmTBIs that escape a symptomatic threshold for a diagnosis of concussion.

In order to understand the pathophysiology at the mild end of the spectrum of human rmTBIs, we carried out biochemical, pathological and behavioral analyses in a mouse model of rmTBI. In this model, mice receive 5 mTBIs (one a day for 5 days) using a cortical impactor to produce head rotations that are approximately 40 times less than the angular accelerations experienced by athletes subsequently diagnosed as concussed. We herein demonstrate that these mTBIs produce pathologies that are transient with apparent resolution as well as pathologies that are late-developing and long-lived including behavioral impairments, pathological tau phosphorylation, metabolomic alterations and white matter ultrastructural abnormalities.

## Material and methods

### Animals

Male C57BL/6 J wildtype mice were purchased from Charles River (Quebec, Canada) at 8–10 w of age. All mice were housed in the same room under identical conditions. They had free access to food and water, except the groups of mice evaluated by touchscreen testing, where food restriction was applied as described below.

### Repetitive Mild TBI

Mice were anesthetized with 3% isoflurane and maintained on 2% isoflurane via a nose cone until immediately after impact. Mice were placed in a Kopf mouse anesthesia mask under a traumatic brain injury device (TBI 0310, Precision Systems and Instrumentation, LLC). Following a 10 mm midline incision, the skin and fascia were reflected. Then animals received a mild controlled cortical impact directly onto the skull, centered on Bregma, with a custom-made, 4 mm-diameter pliant silicone tip. The device was programmed to impact at a depth of 1.0 mm at a velocity of 3.5 m/s with a 500 ms dwell time. These injury depths and speeds are similar to the injury parameters used by others studying mTBI in mice [33, 74]. Mice received 5 impacts (one impact a day for 5 days). Sham mice received 5 min of anesthesia and a scalp incision. Control mice used in the imaging studies and the subsequent immunohistochemistry and blood metabolomics analyses were naïve.

### High-speed videography and kinematic analysis

For kinematic analysis, an independent cohort of 4 mice was subjected to a mTBI and the impact events were recorded at 10,000 frames per second using a high-speed video camera (Fastcam SA6, Photron Limited, Tokyo, Japan). During video recording, we placed the camera on a stable table that is separated from the impact device to avoid vibrations induced by the impact [60]. Head motion was tracked using two markers tightly glued to the right side of the mouse’s shaved head. Velocities and accelerations were determined by discrete differentiation of the position data using in-house developed MATLAB codes. Resultant linear velocity and acceleration were calculated as the magnitude of their respective X and Y components. Linear kinematic parameters were calculated by tracking the markers. Angular rotation of the head during mTBI was determined by the angle of the line joining two marks. Energy transferred from the piston to the head was determined using the equation KE = 0.5 × Me × ΔV^2^, where Me is the effective mass and is approximated by head mass (3.4 g) and ΔV is a change in head velocity. A scaling factor λ [λ = (mass of human brain/mass of mouse brain)^1/3^ = 13.8] was used to estimate the human head-equivalent kinematic parameters from the animal data [79].

### Tissue preparation for light microscopy

Mice were anesthetized with ketamine/Xylazine (2:1) and then underwent trans-cardiac perfusion with ice-cold saline, followed by 4% paraformaldehyde in phosphate-buffer saline (PBS). Brains were post-fixed overnight, infiltrated with sucrose, and embedded in optimal cutting temperature (OCT) medium. For silver staining, thirty floating 40 μm thick coronal cryostat sections were collected at the level of the corpus callosum directly beneath the impact site, and stored in 8% sucrose with 0.1% NaN3 in PBS at 4 °C. For immunohistochemistry, the rest of the brain was cryosectioned at 16 μm and collected serially onto Superfrost Plus slides and stored at − 80 °C.

### Silver staining

Silver staining for diffuse axonal injury (DAI) was focused on the corpus callosum as other models of mTBI have shown intense silver staining in this white matter structure [75]. The 40 μm thick coronal sections from the corpus callosum described above were collected at 48 h, 1 w, 4 w and 10 w post-injury and transferred from the 8% sucrose solution to 4% paraformaldehyde and incubated at 4 °C for at least 7 days. Then silver staining was performed using the FD NeuroSilver Kit II (FD NeuroTechnologies, Ellicott City, MD) according to the manufacturer’s instructions.

### Immunohistochemistry

Immunohistochemistry was focused on the prefrontal cortex (PFC) and hippocampus as our behavioral analyses were specifically focused on tests of attentional control and spatial cognition, cognitive functions controlled by the PFC [51] and the hippocampus [77], respectively. Cryosections (16 μm thick) were rinsed in 0.1 M PBS and blocked in 5% goat serum with 0.1% Triton X-100 for 2 h at room temperature. The sections were then incubated with primary antibodies (diluted in 5% goat serum, Table 1) at 4 °C overnight. The Vectastain Elite ABC kit (Vector laboratories, Burlingame, CA) was used to detect APP immunostaining and visualized with the 3′ 3 diaminobenzidine (DAB, Vector laboratories) chromogen. For fluorescent immunohistochemistry, a donkey anti-mouse IgG, conjugated with Alexa Fluor 488 (1:1000; Invitrogen, Rockford IL, USA) or a goat anti-rabbit IgG, conjugated with Alexa Fluro 594 (1:1000; Invitrogen) were used. Digital images were captured using an Olympus BX41 microscope.

**Table 1:** Primary antibodies

Primary antibody	Host	IHC	Western Blot		Company
		Dilution	Size (KD)	Dilution	
GFAP	Mouse	1:500	51	1:2000	MilliporeSigma
Iba1	Rabbit	1:200	18	1:1000	Abcam
IL-6	Rabbit	/	24	1:1000	Cell Signaling
TNFα	Goat	/	25	1:1000	R&D systems
APP	Rabbit	1:500	100	1:1000	Invitrogen
Synaptophysin	Rabbit	/	38	1:1000	Cell Signaling
PSD-95	Rabbit	1:100	95	1:1000	Invitrogen
Gephyrin	Mouse	1:100	93	1:500	Santa Cruz
Beta-actin	Mouse	/	42	1:5000	Sigma
AT8	Mouse	1:100	/	/	Thermofisher

### Western blotting

Mice used for Western blotting were transcardially perfused with ice cold saline. The prefrontal cortex (PFC) and hippocampus of each mouse were dissected free from the rest of the brain guided by the Allen mouse Brain Atlas. We defined the PFC to include cortex from the oribital area to 1 mm caudal to the bregma, which included the secondary motor cortex; the anterior cingular area, the prelimbic area, the infralimbic area and the agranular insular area as defined by Carlen [14]. Brain tissue was homogenized in RIPA lysis buffer with complete proteinase inhibitor cocktail (Invitrogen). Lysates were clarified by centrifugation at 6000 rpm for 20 min at 4 °C and the supernatants taken for analysis. Protein concentrations in each extract was determined using the BioRad DC protein assay and equal amounts of protein (20 μg/lane) were resolved by SDS–polyacrylamide gel electrophoresis (SDS-PAGE) and electroblotted to Millipore Immobilon-FL PVDF membranes. Blots were incubated in Intercept (TBS) Blocking Buffer (Li-Cor Bioscience, Lincoln, NE) for 2 h at room temperature. Primary antibodies listed in Table 1 were diluted in blocking buffer with 0.1% Tween 20. The blots were incubated with primary antibodies overnight at 4 °C on a shaker. Blots were subsequently washed 3 times for 10 min in TBS/0.2% Tween 20 and incubated with corresponding fluorescent-conjugated secondary antibodies, either the IRDye 800CW Donkey anti-Goat IgG, or the IRDye 800CW Doney anti-Mouse IgG or the IRDye 680CW Doney anti-rabbit IgG (Li-Cor Bioscience), diluted (1:15,000) in blocking buffer with 0.1% Tween 20 and 0.01% SDS for 1 h at room temperature in the dark. The blots were washed 3 times for 10 min in TBS/0.2% Tween 20, followed by a brief rinse in TBS to remove residual Tween 20. Membranes were imaged using the Odyssey Imaging System (LI-COR Biosciences). The relative optical densities of target protein bands within the same gel were quantified using the Image Studio and normalized to β-actin. The same control protein extracts were run on different gels to allow for quantification of protein levels across gels. The shams used for Western blot analyses were sacrificed at 48 h after the sham procedure.

### Imaging

All imaging and spectroscopy were performed in the Centre for Functional and Metabolic Mapping at Western University on a Bruker (Billerica, MA, USA) Avance III HD console interfaced to an Agilent (Santa Clara, CA, USA) 9.4 T small-bore MRI magnet using Agilent Millipede MP30 or MP40 quadrature transmit/receive radio frequency coils. Diffusion Tensor Imaging (DTI) was acquired using a spin-echo acquisition sequence (TE = 36 ms, TR = 1 s, max b-value = 1085 s/mm^2^, 12 gradient directions, FOV = 18.75 mm, 128 × 128 matrix, 31–0.5 mm thick slices). A 2D fast spin-echo anatomical image (TR/TE = 4000/10 ms; FOV = 19.2 × 19.2 mm^2^; matrix = 128 × 128; slice thickness = 0.5 mm) was acquired for tissue/CSF segmentation. Magnetic resonance spectra were acquired from a 2 × 6 × 3 mm^3^ voxel encompassing both hippocampi using VAPOR water suppression [105] and the localization by adiabatic selective refocusing (LASER) pulse sequence (TR/TE = 3250/18.9 ms; averages = 128/8; HS2 R15 adiabatic full passage pulses; bandwidth = 6000 Hz) [30]. Acquisition of a macromolecule only spectra was interleaved with the acquisition of the full spectrum using a single-inversion recovery technique as previously described [46] to remove the contribution of macromolecules to the spectrum in post-processing.

DTI images were pre-processed using fMRI Software Library (FSL, v.5.0.10, Oxford, UK). Images were first registered to a C57BL/6J atlas using a linear transformation (FLIRT) [43] followed by a non-linear registration (FNIRT) in FSL. The resulting transformation matrices were then inverted and used to bring masks of the corpus callosum and hippocampus back into the diffusion space of each subject, where mean diffusion parameter values for each region were then extracted. Mean region of interest (ROI) analysis was performed and focused on two relevant regions of interest: the corpus callosum and the hippocampus. Within each ROI the mean fractional anisotropy (FA), mean diffusivity (MD), axial diffusivity (AD), and radial diffusivity (RD) values were extracted.

All spectra were lineshape and eddy current corrected using combined QUALITY deconvolution (400 points) and eddy current correction [5]. Macromolecules were then fitted using a Hankel singular value decomposition (HSVD) [107] and subtracted from the metabolite spectrum. Using in-house analysis software, suppressed (post macromolecule subtraction) and unsuppressed spectra were fitted in the time domain using prior knowledge produced by density matrix simulations [111] incorporated into a Levenberg–Marquardt minimization routine [6]. Metabolite absolute concentrations and ratios relative to creatine (Cr) were calculated in this study. Absolute concentrations incorporated a correction to account for tissue partial volume (Tissue and CSF) obtained from segmenting the 2D fast spin-echo anatomical image. Metabolite ratios relative to Cr were also calculated, to eliminate the uncertainty associated with partial volume and relaxation corrections from influencing the results.

### Touchscreen behavioral testing

All tasks in the touchscreen battery are motivated by strawberry milkshake reward, and the majority of the tasks require instrumental responses to the touchscreen. Therefore, to provide sufficient motivation, animals are subject to mild food restriction before task training, i.e. ≈ 2.5 g food pellets/day and free access to water. Touchscreen training was performed as previously described [39].

### Paired visual discrimination task with reversal (PVD-R)

Touchscreen testing of behavioral flexibility was based on a visual discrimination and reversal task as previously described [39, 86]. We used a “marble-fan” pair in our testing, where marble was the S+, and fan was the S−. When the mouse touched the S+ (correct), the stimuli were removed, and a reward was delivered along with illumination of the magazine light and a tone. When the animal reached more than 80% correct choices for 2 days in a row, acquisition criterion had been reached. Once all animals in a cohort had reached the acquisition criterion, they were put back on the task and received 2 further task sessions that served as baseline performance (B1 and B2). The animals were then randomized to either the sham or rmTBI group. After the fifth mTBI or sham procedure one-week of recovery was allowed with one maintenance session. The animals were then tested for 2 sessions with the same reward contingencies (B3 and B4). The following day, contingencies of S+ and S− were reversed. The animals were tested for 30 trials per session, for 10 sessions. Following 10 task sessions, the animals then received one maintenance session once a week until the 6th week post-injury, when all the animals were put back again on the task and received 2 task sessions with the previous contingencies (S+: fan; S−: marble). The results were recorded as performance baseline for the 6-week time point (B1 and B2). The following day, contingencies of S+ and S− were reversed again, same as the settings at the acquisition stage. Because the mice needed a large number of correction trials after the reversal, they received 10 trials per session for the first 3 days (R1–3), followed by the regular 30 trials per session for 10 more sessions (R4–13). The percentage of accuracy was recorded and compared between the sham and injured animals.

### 5-Choice serial reaction time task (5-CSRTT)

The general procedure for the 5-CSRTT in a touchscreen-based automated operant system for mice was described previously [87]. Mice were trained on this task until they reached a criterion of stable performance at 80% accuracy with stimuli duration of 2 s. After reaching this criterion they were randomized to receive either five mTBIs or five sham procedures. There was no difference in performance on the 5-CSRTT between groups prior to the experimental procedure (sham mice versus rmTBI mice). Probe trials at 2, 6 and 10 w post-injury were performed by increasing the attentional demand of the task by decreasing the stimulus duration using 1.5, 1.0, 0.8 and 0.6 s stimuli lengths. The percentage of accurate trials was recorded and compared between sham and injured mice.

### Morris water maze

Sixteen weeks after the last mTBI, all the mice were trained in the standard Morris water maze with a hidden platform (Noldus, Wagneningen. the Netherlands). Thirteen sham and 15 rmTBI mice were tested over 4 days (4 trials/session). A transparent escape platform was placed 1 cm below the water surface in a fixed position (Q4, SW). In each trial, mice were placed at one of the starting locations in a random order and were allowed to swim until they located the platform. Mice failing to find the platform within 60 s were placed on it for 15 s. In the probe session, the platform was removed from the target quadrant, and mice were allowed to swim for 60 s. All the trials were recorded and traced with an image tracing system (ANYMAZE, Stoelting Co.), connected to a video camera placed above the pool. The time each mouse spent in the target quadrant was compared to the average time spent in the 3 non-target quadrants.

### Metabolomics

For metabolomics analyses blood was collected from the right atria of mice just prior to cardiac perfusion. Approximately 400 μl of whole blood was withdrawn using an insulin syringe. Blood samples were then immediately transferred into a vacutainer tube (BD #367820) and incubated at room temperature for 60 min to allow clotting. Blood samples were then centrifuged for 15 min at 2000 g at 4 °C. The supernatant (serum) was collected and stored at − 80 °C. Serum samples from shams and injured mice at 48 h, 1, 4 and 10 w post-injury were analyzed for their levels of 144 different metabolites by direct flow injection and LC–MS/MS compound identification and quantification (DI/LC–MS/MS) carried out by the Metabolomics Innovation Center (TMIC) at the University of Alberta using the Biocrates kit. Reliable levels on 144 different metabolites were returned (36 of the 144 metabolites in the screen were undetectable in our samples). We applied a targeted quantitative metabolomics approach to analyze the serum samples using a combination of direct injection mass spectrometry (Absolute*IDQ*™ Kit) with a reverse-phase LC–MS/MS Kit. The Kit is a commercially available assay from BIOCRATES Life Sciences AG (Austria). This kit, in combination with an ABI 4000 Q-Trap (Applied Biosystems/MDS Sciex) mass spectrometer, can be used for the targeted identification and quantification of different endogenous metabolites including amino acids, acylcarnitines, biogenic amines, glycerophospholipids, sphingolipids and sugars. The method used combines the derivatization and extraction of analytes, and the selective mass-spectrometric detection using multiple reaction monitoring (MRM) pairs. Isotope-labeled internal standards and other internal standards are integrated in Kit plate filter for metabolite quantification. The Absolute*IDQ* kit contains a 96 deep-well plate with a filter plate attached with sealing tape, and reagents and solvents used to prepare the plate assay. First 14 wells in the Kit were used for one blank, three zero samples, seven standards and three quality control samples provided with each Kit. All the serum samples were analyzed with the AbsoluteIDQ kit using the protocol described in the AbsoluteIDQ user manual. Briefly, serum samples were thawed on ice and were vortexed and centrifuged at 13,000×g. 10 µL of each serum sample was loaded onto the center of the filter on the upper 96-well kit plate and dried in a stream of nitrogen. Subsequently, 20 µL of a 5% solution of phenyl-isothiocyanate was added for derivatization. After incubation, the filter spots were dried again using an evaporator. Extraction of the metabolites was then achieved by adding 300 µL methanol containing 5 mM ammonium acetate. The extracts were obtained by centrifugation into the lower 96-deep well plate, followed by a dilution step with kit MS running solvent. Mass spectrometric analysis was performed on an API4000 Qtrap^®^ tandem mass spectrometry instrument (Applied Biosystems/MDS Analytical Technologies, Foster City, CA) equipped with a solvent delivery system. The samples were delivered to the mass spectrometer by a LC method followed by a direct injection (DI) method. The Biocrates MetIQ software was used to control the entire assay workflow, from sample registration to automated calculation of metabolite concentrations to the export of data into other data analysis programs. A targeted profiling scheme was used to quantitatively screen for known small molecule metabolites using multiple reaction monitoring, neutral loss and precursor ion scans.

### Ultra high performance liquid chromatography (UHPLC) used for metabolomics analyses

An Agilent 1260 series UHPLC system (Palo Alto, CA, USA) and an Agilent reversed-phase Zorbax Eclipse XDB C18 col-umn (3.0 mm × 100 mm, 3.5 μm particle size, 80 Å pore size) with a Phenomenex (Torrance, CA, USA) SecurityGuard C18 pre-column (4.0 mm × 3.0 mm) were used for all online LC/DFI-MS/MS analyses with an AB Sciex QTRAP^®^ 4000 mass spectrometer (Concord, ON, CA). The controlling soft-ware for the sample analysis was Analyst^®^ 1.6.2. The UHPLC parameters used to analyze amino acids and biogenic amines were as follows: solvent A 0.2% (v/v) formic acid in water, and solvent B 0.2% (v/v) formic acid in acetonitrile. The gradient profile for this UHPLC solvent run was as follows: t = 0 min, 0% B; t = 0.5 min, 0% B; t = 5.5 min, 95% B; t = 6.5 min, 95% B; t = 7.0 min, 0% B; and t = 9.5 min, 0% B. The column oven was set at 50 °C. The flow rate was 500 μL/min, and the sample injection volume was 10 μL. The mass spectrometer was set to a positive electrospray ionization mode with a scheduled multiple reaction monitoring (MRM) scan. The IonSpray voltage was set at 5500 V and the tem-perature at 500 °C. The curtain gas (CUR), ion source gas 1 (GAS1), ion source gas 2 (GAS2) and collision gas (CAD) were set at 20, 40, 50 and medium, respectively. The entrance potential (EP) was set to 15 V. Likewise, the declustering potential (DP), collision energy (CE), collision cell exit poten-tial (CXP), MRM precursor ion (Q1) and fragment ion (Q3) were set individually for each analyte and isotope-labelled ISTD. For DI-MS/MS analysis of acylcarnitines, lipids and glucose, the UHPLC autosampler was connected directly to the MS ion source by red PEEK tubing. The FIA running buffer mentioned above was used as the mobile phase while the flow rate was programmed as follows: t = 0 min, 30 μL/min; t = 1.6 min, 30 μL/min; t = 2.4 min; 200 μL/min; t = 2.8 min, 200 μL/min; and t = 3.0 min, 30 μL/min. The sample injection volume was 20 μL. The mass spectrometer was set to a positive electrospray ionization mode with MRM scanning to analyze lipids and acylcarnitines, and to a negative electrospray ionization mode to detect glucose/hexose. The IonSpray voltage was set at 5500 V for the positive mode and − 4500 V for the negative mode, whereas the temperature was set at 200 °C for both polarities. The CUR, GAS1, GAS2 and CAD were set at 20, 40, 50 and medium, respectively. The EP and CXP were set at 10 V and 15 V separately for positive mode; and − 10 V and − 15 V respectively for negative mode. Likewise, the DP, CE, Q1 and Q3 were set individually for each analyte and ISTD.

### Electron microscopy (EM)

Mice designated for EM studies were perfused with 0.1 M phosphate buffer, followed by 4% paraformaldehyde supplemented with 2% glutaraldehyde. Brains from sham and rmTBI mice (N = 6 per group) were sectioned in the sagittal plane at 100 μm using a vibratome (Leica VT1000S). Sections at the midline were collected in 24-well plates filled with 0.1 M phosphate buffer. In addition, two brains from each group were sectioned in the coronal plane at 100 μm. Further processing was carried out as previously described [59]. Briefly, the specimens were post-fixated with 1% Osmium Tetroxide in 0.1 M Cacodylate buffer for 1 h followed by overnight en-bloc staining with 1% Uranyl Acetate (at T = 4 °C). Brain tissues were first rinsed with double-distilled water and then dehydrated in an ascending series of ethanol solutions and embedded in Spurr’s resin between two Aclar films at 60 °C for 2–3 days for polymerization. After polymerization, the Genu of the corpus callosum was isolated using a stereomicroscope [59]. Semi-thin sections (0.5 μm thick) were generated using an ultramicrotome (Ultramicrotome Reichert-Jung Ultracut E; Leica Microsystems, Wetzlar, Germany). The sections were transferred to a SuperFrost slide and stained with 1% toluidine blue for evaluation of area of interest within corpus callosum by light microscopy. Ultrastructural myelin abnormalities were observed using the Philips CM10 TEM (Philips Electronics, Eindhoven, The Netherlands) at the Biotron facility at Western University.

### Electron microscopy measurements of myelin thickness and axonal counts

Photomicrographs of sections through the corpus callosum were taken at 5800 × magnification. Eight to twelve images per animal were quantified. To measure the myelin thickness, we used Image Pro Premium software (Media Cybernetics, Rockville MD). A binary mask was first created using the minimum variance “auto dark” algorithm to select areas of positive myelin staining. Then, using the line profile function, two horizontal lines and two vertical lines were created and applied onto the same locations of each image mask. Only axons that fell on one of the four lines were evaluated. A total of 824 ± 136 axons were counted per animal. With the edge function, the rising length and falling length were recorded separately and sequentially. Here, we created a macro to standardize and automate the measurement of each image. The thickness of a myelin sheath was then calculated as T_myelin_ = L_falling _− L_rising_. The numbers myelinated axons and demyelinated axons were counted manually. Demyelinated axons were identified as having a diameter larger than 0.3 μm with no detectable compact myelin [71, 103]. Axons smaller than 0.3 μm were excluded from analysis as potentially being normal unmyelinated fibers.

### Experimental timeline

154 male mice in total were used in these experiments, 4 naïve, 34 shams, 16 controls, and 100 rmTBI mice. Two types of control mice were used in these experiments: Shams, generated by subjecting mice to 5 min of anesthesia and a scalp incision once a day for 5 days in a row, and naïve controls, hereafter referred as controls. As outlined in Table 2, the experimental plan consisted of 5 experimental groups of mice. Group 1 mice were used for head kinematics of the mTBI. Group 2 mice were used for Western blot analyses and was comprised of sham mice (sacrificed 48 h after the last sham procedure) and rmTBI mice sacrificed at the times indicated. Group 3 mice were used for imaging (MRS and DTI) at the times indicated and then were sacrificed for blood metabolomic and immunohistochemistry analyses. The Group 3 control mice were naïve until they were imaged (requiring ~ 3 h of isoflurane exposure) and then 1 week later underwent blood collection for metabolomic analyses before being sacrificed and processed for immunohistochemistry. We reasoned that the short isoflurane exposures (~ 5 min) that occurred during the sham procedures would have minimal effects compared to the hours long isoflurane exposure incurred by the control and rmTBI mice at the time of imaging. Supporting this contention, Western blot analyses comparing PFC protein extracts between controls and sham mice 48 h after the last of the 5 sham procedures revealed no differences between these animals. The group 3 mice were approximately 4–5 months old when imaged and sacrificed for metabolomics analyses. Group 4 mice consisting of shams and rmTBI mice were evaluated for attention (5-CSRTT) and provided the biological samples for the EM analyses. Group 5 mice consisting of shams and rmTBI mice underwent testing for behavioral flexibility (PVD-R) and for spatial cognition (Morris water maze). Within group 5 seven sham and 3 rmTBI mice were excluded from analyses due to health concerns that arose during the touchscreen testing.

**Table 2:** Experimental timeline

Group #	Tests	Sample size (n) for shams and rmTBI mice at each time point	Time points
I	mTBI kinematics	4 control	
II	Western Blot	6 sham (48 h after the last sham procedure) and 6 rmTBI	8 h, 48 h, 1 w and 20 w
IIa	Western blot (Naïves and Shams)	4 naïve, 5 sham (8 h after the last sham procedure) and 5 shams (48 h after the last sham procedure)	8 h, 48 h
III	DTI and MRS/ IHC/ Metabolomics	12 control and 12 rmTBI for MRS, DTI and metabolomics; 6 control and 6 rmTBI for IHC;	48 h, 1 w, 4 w and 10 w
IV	5-CSRTT	8 sham, 10 rmTBI	2 w, 6 w and 10 w
	EM	6 sham, 6 rmTBI	20 w
V	PVD	14 sham, 15 rmTBI	2 w, 6 w and 10 w
	MWM	13 sham, 15 rmTBI	16 w

### Statistics

Graphpad Prism 8.0 was used for statistical analyses and graphing of quantitative data. Data is expressed as mean ± SEM. The sample size was predetermined from prior experiments as n of 6 mice per condition for quantification with western blotting analysis, and myelin changes of corpus callosum in EM pictures. A one-way ANOVA was performed to determine significant differences across multiple post-injury time points. For the touchscreen behavioral tests, we pre-determined the sample size using power analysis (Gpower 3.1). The specific sample size and statistical tests used for each dataset are noted in the figure legends. Unpaired student *t*-test was used to compare rmTBI and sham results collected in the water maze probe test. Two-way ANOVA was used to analyze the data from touchscreen tests, where two animal groups were tested with multiple conditions; as well as the latency studies, where rmTBI mice were compared to sham at multiple time points. Tukey’s multiple comparison test was performed as post hoc test for both one-way and two-way ANOVA. Statistical significance was determined as *p* ≤ 0.05.

## Results

### Head kinematics of mTBI

**Fig. 1:**
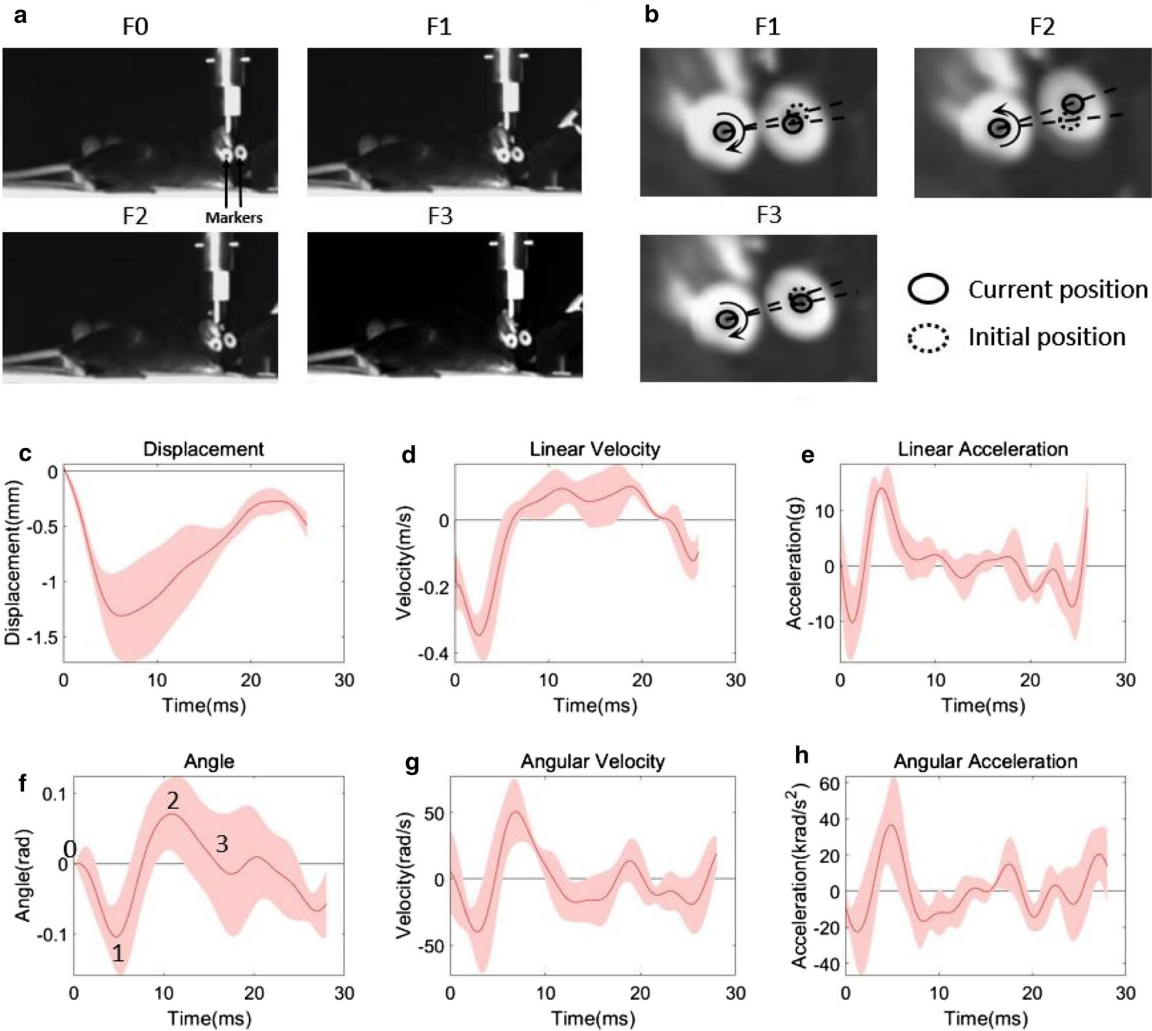
Head kinematic during mTBI. **a** Head motion was tracked by high-speed videography using two markers. **b** The pictures indicate the head position during impact filmed at frames F1, F2 and F3. F0 is a frame of the mouse’s head before impact. The mouse head shows flexion right after impact (frame F1 in a and mark 1 in graph f), and then shows slight extension (frame F2 in a and mark 2 in graph f), followed by a slight flexion (frame F3 in a and mark 3 in c-e and f–h, respectively. Data were presented as mean ± standard deviation. Standard deviation was presented as shadow. Bar = 10 μm

Changes in head kinematics during an impact were assessed using high speed videography (Fig. 1a) as we have previously described [60]. The average peak linear displacement was 1.3 mm ± 0.4 mm (Fig. 1c). The average peak linear velocity and linear acceleration were 0.35 ± 0.07 m/s and 14.0 ± 2.2 g, respectively (Fig. 1d, e). The average peak angular velocity and angular acceleration were approximately 50.6 ± 24.6 rad/s and 36.6 ± 24.5 krad/s^2^ (Fig. 1f). These linear and angular accelerations were far lower than the linear and angular accelerations measured by others studying TBI using the CHIMERA model in mice [75] or the weight drop model of mTBI in rats [41]. Furthermore, when using a scaling factor of *λ* = (mass of human brain/mass of mouse brain)^1/3^ = 13.8 [79], the mTBI used in the present study is estimated to produce forces in the mouse brain that are 40-fold less than or those produced in the brains of athletes experiencing concussion [88]. All subsequent experiments used these parameters to repeat the injury once a day for 5 consecutive days.

### rmTBI induces diffuse axonal injury

**Fig. 2:**
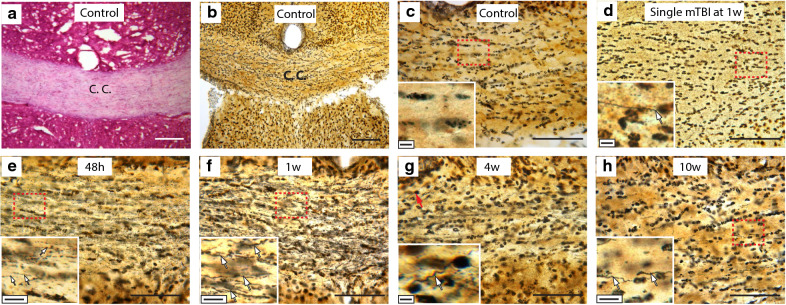
Diffuse axonal injury in corpus callosum (CC) after rmTBI. **a** H&E staining indicated the corpus callosum area. While background silver-staining of nuclei was visible in all sections, silver-stained fibers were not observed in the sections from control mice (**b**, **c**). Mice after a single mTBI (**d**) showed very few, if any, silver-stained fibers. In sections from rmTBI mice silver-stained fibers with the punctate beading typical of DAI were easily detected at 48 h, 1w, 4w and 10w after injury (**e**–**h**). Scale bar = 50 μm. Insets are high magnifications of the red-boxed areas of the panel. Inset image shows prominent silver-stained “cork-screw” shaped axons (arrows). Scale bar in insets in c-f = 20 μm

Diffuse axonal injury (DAI) is a common neuropathological consequence of mTBI [45]. A primary outcome of DAI is the disruption of axonal transport that results in the accumulation of degenerating cellular materials into swellings that are detectable by silver staining [45]. To evaluate whether our rmTBI injury caused DAI, we carried out silver staining of histological sections of the brains of rmTBI mice at 48 h, 1, 4 and 10 w post-injury. Examination of the silver-stained sections revealed strong silver staining in the corpus callosum at all time points analyzed (Fig. 2e–h). High power magnification revealed that the silver-stained axons had the typical beaded appearance associated with DAI [45]. Brain sections from control mice failed to show any silver-stained axons while mice one week after a single mTBI revealed the occasional axon with positive silver staining (Fig. 2b–d).

**Fig. 3:**
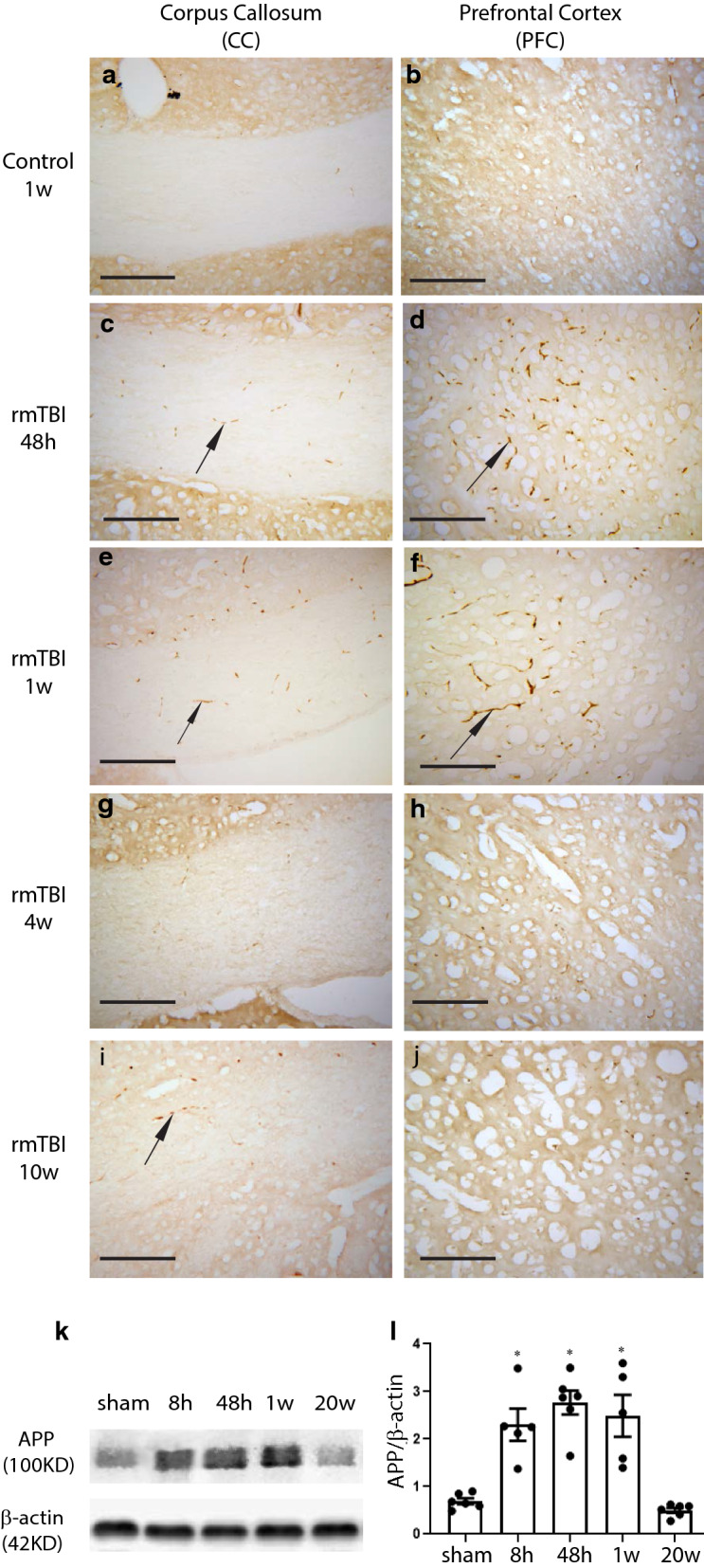
Amyloid precursor protein (APP) is upregulated following rmTBI. **a**–**j** Representative immunohistochemical photomicrographs demonstrate APP expression (arrows) in the corpus callosum and prefrontal cortex, in control sections (**a**, **b**) and at 48 h (**c**, **d**), 1 w (**e**, **f**), 4 w (**g**, **h**) and 10 w (**i**, **j**) post-rmTBI, respectively. Scale bar = 50 μm. (**k**, **l**) Western blot and densitometric analyses of full-length APP in the cortex of sham mice, 48 h after the last sham procedure, and in rmTBI mice at 8 h, 48 h, 1 w and 20 w post-rmTBI, demonstrate a significant increase in APP levels at 8 h, 48 h and 1 w, post injury that resolves to baseline levels at 20 w post injury. β-actin levels were used as a protein loading control. Asterisk indicates statistically different from shams, *p* < 0.05; one-way ANOVA

Immunohistochemistry to detect axonal accumulations of amyloid precursor protein (APP) has also been used as a measure of DAI [35, 85, 95]. APP accumulates in damaged axons within hours of an injury and when compared to silver staining, APP immunoreactivity reveals greater degrees of axonal pathology [32]. Thus we evaluated APP immunoreactivity in brain sections of the rmTBI mice. While sections from controls (Fig. 3a, b) failed to show significant levels of APP immunoreactivity, APP accumulations were easily visible in the PFC and corpus callosum of rmTBI mice at 48 h and 1 w post-injury (Fig. 3c, f). Studies have shown that APP not only accumulates at sites of axonal injury, but that its expression levels also increase after TBI [84]. Western blot analyses of protein extracts from mTBI mice demonstrated significantly increased levels of APP protein in the PFC of injured mice at 8 h, 48 h and 1 w post-injury compared to shams (Fig. 3k, l).

### rmTBI induces transient gliosis and inflammation

**Fig. 4:**
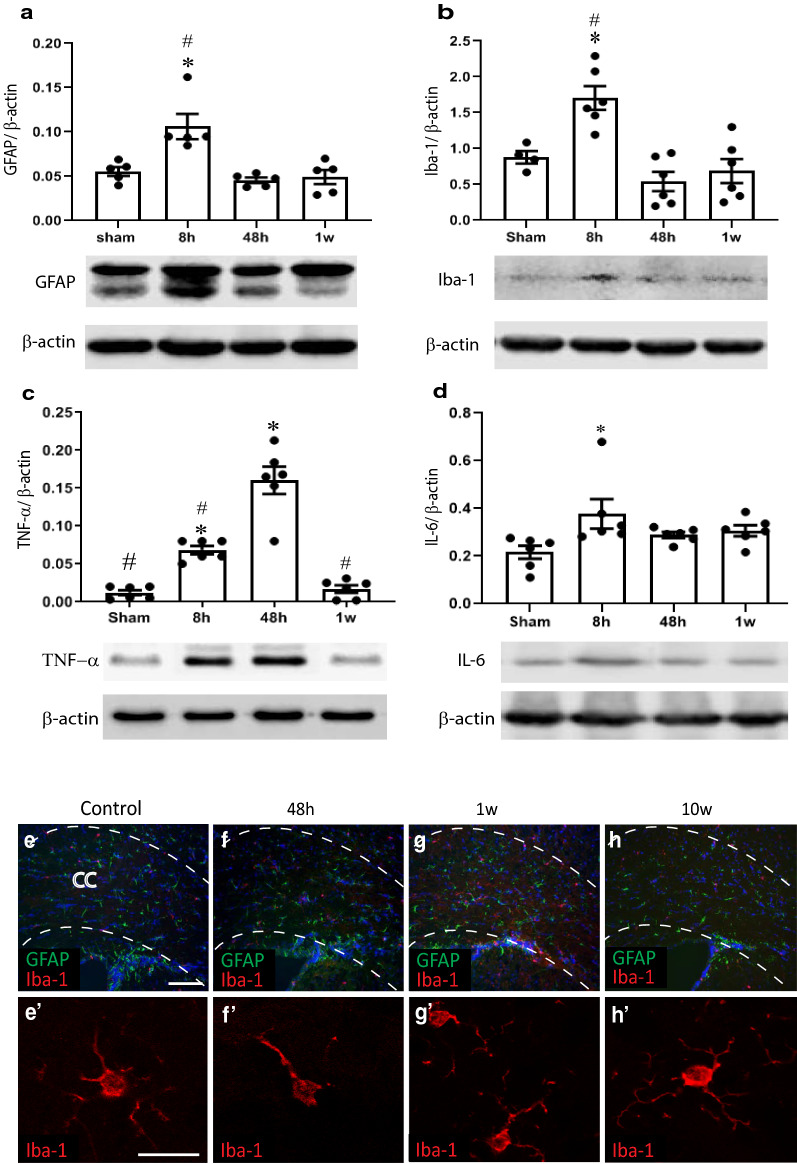
Repetitive mTBI leads to a mild inflammatory response. Western blot and subsequent densitometric analyses show time-course of GFAP (**a**), Iba1 (**b**), TNFα (**c**) and IL-6 (**d**) levels in the prefrontal cortex from mice 48 h after the last sham procedure and from mice 8 h, 48 h and 1 week post-injury. β-actin levels were used as a loading control. (Asterisk indicates statistically different from shams, and # indicates statistically different from rmTBI 48 h, *p* < 0.05; one-way ANOVA). Representative immuno-fluorescent stained photomicrographs showing GFAP and Iba1 expression in the corpus callosum in controls (**e**), and in mice at 48 h (**f**), 1 week (**g**) and 10 w (**h**) post-rmTBI. Scale bar = 50 μm. Higher magnification images of Iba1 immunostaining to better demonstrate microglial morphology at the corresponding time points are shown in the insets (**e**′–**h**′). Scale bar in e’–h’ = 20 μm

The astrocytic and inflammatory responses to rmTBI were assessed by a combination of immunohistochemistry and Western blot analyses. We previously demonstrated that a protracted astrogliosis and microgliosis ensues following repeated mild lateral fluid percussion injury that persists for months as measured by immunohistochemistry and Western blot analyses [97]. The repeated mTBI injury used here produced a twofold transient increase in GFAP expression at 8 h after injury compared to shams that returned to baseline levels by 48 h (Fig. 4a). Iba1 levels, a marker of microglial cells [22], was also elevated at 8 h after the last of the 5 mTBIs as assessed by Western blot analyses and returned to baseline levels by 48 h (Fig. 4b). Immunohistochemistry using Iba1 antibodies demonstrates that while microglia in sham controls have the typical ramified appearance of resting microglia, at 48 h after the last of the five mTBIs, the microglia in injured mice have taken on a more rounded and “bushy” appearance that returns to a baseline resting appearance by 1 week post-injury (Fig. 4E-H). The levels of pro-inflammatory cytokines TNFα (tumor necrosis factor α) and IL-6 (interleukin-6) in the PFC were similarly found to be elevated only acutely (at 48 h and 8 h) after the last of the 5 mTBIs as evaluated by Western blots (Fig. 4c, d). The increases in the levels of GFAP, Iba1, TNFα and Il-6 in the protein samples taken from rmTBI mice were identified by comparing them to the levels of GFAP, Iba1, TNFα and Il-6 in the protein samples from shams 48 h after their last sham procedure. Western blot analyses of protein samples from the brains of naïve mice, shams at 8 h after their last sham procedure and shams at 48 h after their last sham procedure demonstrated no difference in the levels of these inflammatory markers between any of these groups (Additional file 1: Fig. S1) attesting to the negligible effects of the sham procedures.

### rmTBI induces MRS and DTI changes

The silver staining of brain sections from the rmTBI mice suggested DAI was present from 48 h through to 10 w after the last of the 5 mTBIs. To determine whether the axonal injury might be accompanied by white matter changes we employed DTI which is sensitive to changes in tissue microstructure and structural connectivity in the brain [1]. Fractional anisotropy (FA) is a DTI measurement considered to be an indication of white matter integrity [1]. The injured mice showed increased FA compared to controls in the corpus callosum and hippocampus at 48 h post-injury and in the hippocampus at 1 w post-injury. Within the rmTBI mice, FA was increased at 48 h post-injury compared to rmTBI mice 4 w and 10 w post-injury in the corpus callosum and hippocampus. These changes in FA were driven by reductions in radial diffusivity (Table 3).

**Table 3:** DTI region of interest analysis

Region of interest	Imaging metric	Control	48 h	1 Week	4 Weeks	10 Weeks	ANOVA (F) or
		(Mean ± SEM)					Kruskal–Wallis (K)
Hippocampus	FA × 10^–1^	3.17 ± 0.108	3.74 ± 0.144*	3.76 ± 0.174*	**3.04 ± 0.109#**	**3.13 ± 0.0844#**	F = 7.54; *p* < 0.0001
	MD × 10^–3^	1.04 ± 0.0227	1.02 ± 0.0261	1.02 ± 0.0340	0.984 ± 0.0277	1.11 ± 0.0422	F = 2.16; *p* = 0.087
	RD × 10^–3^	0.892 ± 0.0163	0.797 ± 0.0204	**0.789 ± 0.0228**^†^	**0.830 ± 0.0245**^†^	**0.936 ± 0.0384#**	F = 6.11; *p* = 0.0004
	AD × 10^–3^	1.42 ± 0.0490	1.43 ± 0.0354	1.42 ± 0.0466	1.29 ± 0.0387	1.45 ± 0.0509	F = 1.92; *p* = 0.12
Corpus Callosum	FA × 10^–1^	3.49 ± 0.0667	3.95 ± 0.0980*	3.66 ± 0.147	**3.43 ± 0.139#**	**3.36 ± 0.0869#**	F = 4.56; *p* = 0.003
	MD × 10^–3^	0.939 ± 0.0203	0.904 ± 0.0189	0.918 ± 0.0162	0.914 ± 0.0189	0.883 ± 0.0137	F = 1.29; *p* = 0.28
	RD × 10^–3^	0.762 ± 0.0169	0.689 ± 0.0763*	0.739 ± 0.0125	**0.746 ± 0.0158#**	0.726 ± 0.0135	F = 4.24; *p* = 0.0048
	AD × 10^–3^	1.29 ± 0.0302	1.30 ± 0.0322	1.26 ± 0.0353	1.25 ± 0.0325	1.22 ± 0.0288	F = 1.07; *p* = 0.38

Bold indicates statistically different according to the following key

*SEM* = standard error of the mean; * = significance from controls; # = significance from 48 h; † = significance from 10 weeks; FA = fractional anisotropy; MD = mean diffusivity; RD = radial diffusivity; AD = axial diffusivity

As we have previously found alterations in MRS derived metabolite levels in concussed and non-concussed female contact athletes [93], we used MRS to evaluate NAA, lactate, glutamate, glutamine, glutathione, creatine, taurine, choline, *myo*-inositol, as well as ratios relative to creatine (X/Cr), using a 36 mm^3^ voxel centered on the midline of the corpus callosum (Table 4). N-Acetyl aspartic acid (NAA) was found to be reduced at 1-week post rmTBI from 48 h post rmTBI (Tukey’s multiple comparisons test *p* = 0.0185), coinciding with the elevated FA measured by DTI. Additionally, altered levels of creatine, taurine, Glu/Cr and Gln/Cr were found at 10 w. Creatine was decreased from controls (Tukey’s multiple comparisons test *p* = 0.0106), while taurine was decreased from controls, 48 h, and 1 w (Tukey’s *p* = 0.0266, *p* = 0.0007, *p* = 0.0128, respectively). Among the ratios at 10 w, Glu/Cr was elevated from controls (Tukey’s *p* = 0.0346) and 1 w (Tukey’s *p* = 0.0495), while Gln/Cr was elevated from 48 h (Tukey’s *p* = 0.0451) (Table 4).

**Table 4:** MRS absolute concentration and ratios

Imaging metric	Control	48 h	1 week	4 weeks	10 weeks	ANOVA (F) or
(Hippocampus)	(Mean ± SEM)					Kruskal–Wallis (K)
NAA	4.1 ± 0.22	4.5 ± 0.31	**3.5 ± 0.17 #**	3.8 ± 0.14	3.7 ± 0.16	F = 3.27; *p* = 0.018
Lactate	2.4 ± 0.17	2.9 ± 0.20	2.4 ± 0.11	2.2 ± 0.12	**2.1 ± 0.21#**	F = 3.39; *p* = 0.016
Glutamate	4.1 ± 0.30	3.5 ± 0.29	3.2 ± 0.13	3.5 ± 0.32	4.2 ± 0.28	F = 2.65; *p* = 0.044
Glutamine	0.39 ± 0.13	0.35 ± 0.15	0.88 ± 0.17	1.0 ± 0.32	1.1 ± 0.15	F = 3.08; *p* = 0.025
Glutathione	1.7 ± 0.20	1.4 ± 0.22	1.5 ± 0.23	1.0 ± 0.17	0.97 ± 0.18	F = 2.2; *p* = 0.082
Creatine	7.4 ± 0.37	7.0 ± 0.47	6.6 ± 0.39	6.1 ± 0.10	5.7 ± 0.30*	F = 3.55; *p* = 0.013
Taurine	9.7 ± 0.83	11 ± 0.61	9.9 ± 0.68	8.7 ± 0.72	6.8 ± 0.49*^,#,†^	F = 5.3; *p* = 0.0011
Choline	1.6 ± 0.11	1.6 ± 0.13	1.8 ± 0.15	1.7 ± 0.17	1.4 ± 0.11	F = 1.13; *p* = 0.35
*Myo*-inositol	1.9 ± 0.45	3.1 ± 0.38	2.5 ± 0.36	3.1 ± 0.51	1.7 ± 0.28	F = 2.66; *p* = 0.043
NAA/Cr	0.61 ± 0.020	0.74 ± 0.049	0.62 ± 0.037	0.70 ± 0.022	0.75 ± 0.047	K = 9.79; *p* = 0.044
Lactate/Cr	0.88 ± 0.13	0.90 ± 0.090	0.84 ± 0.074	0.82 ± 0.076	0.59 ± 0.093	F = 1.69; *p* = 0.17
Glutamate/Cr	0.81 ± 0.11	0.83 ± 0.099	0.83 ± 0.056	0.91 ± 0.083	1.2 ± 0.10*^,¥^	F = 3.02; *p* = 0.026
Glutamine/Cr	0.12 ± 0.047	0.086 ± 0.037	0.20 ± 0.042	0.25 ± 0.078	**0.28 ± 0.045#**	F = 2.86; *p* = 0.033
Glutathione/Cr	0.89 ± 0.076	0.75 ± 0.11	0.82 ± 0.11	0.59 ± 0.10	0.60 ± 0.082	F = 1.82; *p* = 0.14
Taurine/Cr	1.8 ± 0.14	2.0 ± 0.12	1.9 ± 0.095	1.8 ± 0.14	1.5 ± 0.11	F = 2.36; *p* = 0.065
Choline/Cr	1.4 ± 0.075	1.4 ± 0.098	1.8 ± 0.15	1.7 ± 0.14	1.7 ± 0.15	F = 2.85; *p* = 0.033
*Myo*-inositol/Cr	0.64 ± 0.13	1.1 ± 0.18	0.76 ± 0.077	1.1 ± 0.15	0.58 ± 0.080	F = 3.17; *p* = 0.021

Bold indicates statistically different according to the following key

SEM = standard error of the mean; * = significance from controls; # = significance from 48 h; ¥ = significance from 1 week; † = significance from 10 weeks; NAA = *N*-acetyl aspartate, Cr = creatine

### rmTBI leads to evidence of synaptic remodeling

**Fig. 5:**
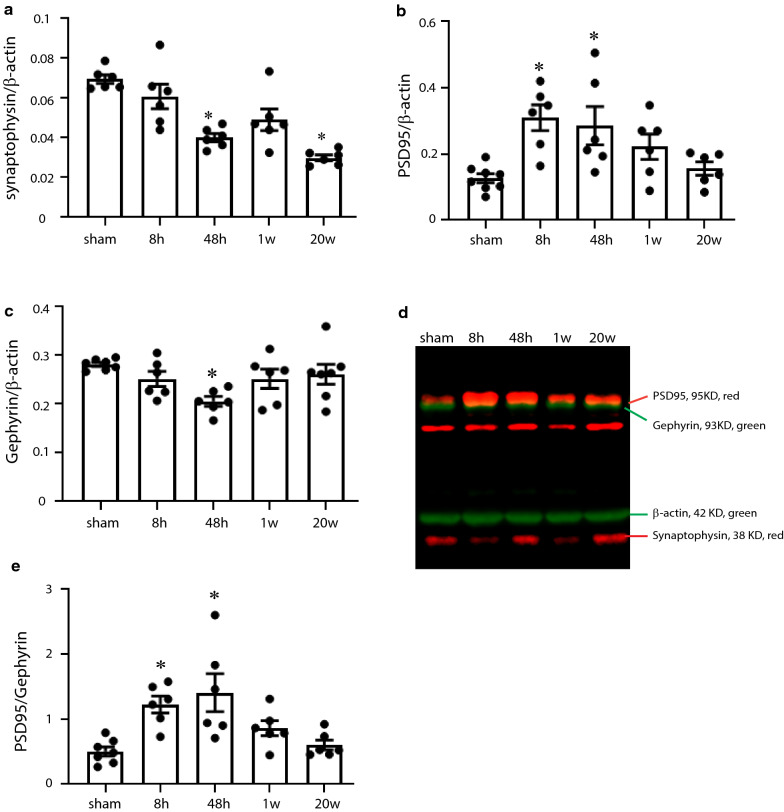
Alterations in synaptic protein levels in the PFC post-rmTBI. Protein extracts from the PFC of sham and rmTBI mice at different time points post-injury were separated by PAGE and the Western blots probed simultaneously with antibodies to detect PSD95, Gephyrin, synaptophysin and β-actin. The relative optical densities of protein bands for **a** synaptophysin, **b** PSD95 and **c** Gephyrin within the same gel were quantified and normalized to β-actin. A representative immunoblot is shown in **d**. The ratios of PSD95: Gephyrin levels was elevated in the PFC of rmTBI mice at 8 h and 48 h post-injury (**e**). Asterisk denotes statistically significant different from shams (48 h after the last sham procedure), *p* < 0.05; one-way ANOVA

The presence of DAI in the injured mice suggested the possibility that a percentage of injured axons may have been sufficiently damaged as to cause axonal disconnection and synaptic loss. To investigate rmTBI mice for possible changes in synaptic proteins Western blot analyses were carried out to evaluate synaptophysin levels in protein extracts from the PFC in sham and rmTBI mice [108]. This analysis showed a significant reduction in synaptophysin levels in brain extracts from injured mice at 48 h, and at 20 w after the last of the 5 mTBIs (Fig. 5A). Since TBI is known to lead to an imbalance in cortical excitation and inhibition [7, 11, 15, 19, 110] we predicted that the changes in synapse number suggested by the reduction in synaptophysin levels might be due to changes in the levels and ratios of excitatory glutamatergic and inhibitory GABAergic synapses. To investigate this possibility we evaluated the levels of PSD95, a marker of glutamatergic terminals [24], and of Gephyrin, a marker of GABAergic terminals [91] in the PFC of sham and injured mice. The western blot analyses showed that PSD95 levels were significantly increased in injured mice compared to shams at 8 h and 48 h after the last of the 5 injuries and that Gephyrin levels were significantly decreased compared to shams at 48 h after the last mTBI (Fig. 5b, c). Thus the ratio of PSD95 levels: Gephyrin levels were significantly elevated in the injured mice at 8 and 48 h after the last injury compared to shams (Fig. 5d). This suggests that at least up to 48 h after the last of the 5 mTBIs an imbalance in the excitatory and inhibitory synapses exists in the PFC of rmTBI mice.

### rmTBI leads to reduced attentional control but does not affect cognitive flexibility

The prominent pathological changes observed suggested the possibility that rmTBI may cause behavioral deficits. Many studies report that mTBI patients demonstrate decreased concentration and slowed reaction times [38, 40, 53] as well as reduced cognitive flexibility [25, 55, 78], high-level cognitive functions that have not been routinely evaluated in mouse models of TBI. Thus, we evaluated rmTBI mice on the PVD-R task that measures behavioural flexibility [13] (dependent on cortical-striatal function) and on the 5-CSRTT that measures attentional control [10] (a cognitive domain dependent on PFC function).

**Fig. 6:**
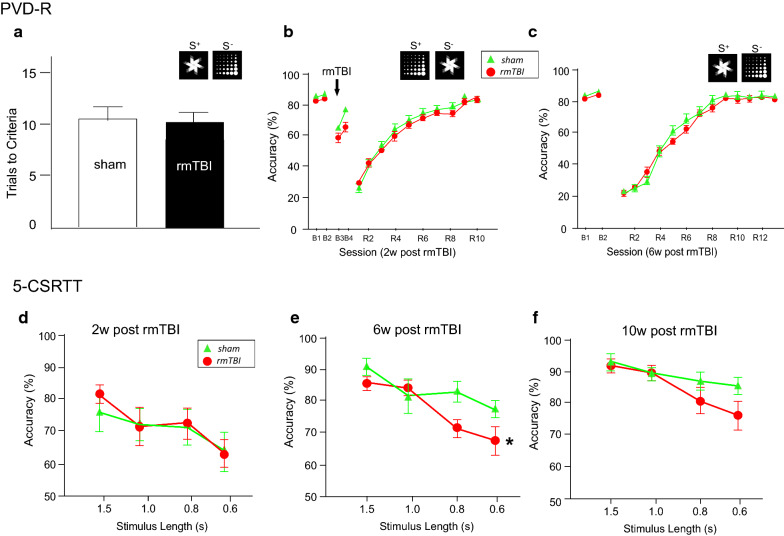
Deficits in attention but not visual discrimination revealed by touchscreen testing after rmTBI. **a**–**c** Paired visual discrimination. **a** Task acquisition: trials required to reach criteria (> 80% correct responses on 2 consecutive days).** b** All the mice received 2 sessions of baseline testing (B1, B2) before being randomly assigned to sham or injury group. After 2-week recovery, the mice were re-baselined for 2 sessions (B3, B4), then preform the reversed task contingencies (the previous S + became S−; and the previous S− the S+) for 10 days (R1–R10). The choice of accuracy was compared between sham and rmTBI. **c** At 6-week post rmTBI, all the mice received 2 sessions of baseline testing. Then the task contingencies were reversed again (back to the original S+ and S−). The mice were tested with 3 mini-sessions (10 trials per day, R1–R3), then followed by full sessions for 10 days (20 trials per day, R4–R13). The performance levels were compared between two groups. n = 15 for rmTBI group; n = 14 for shams. d-f 5-choice Serial Reaction Time Testing (5-CSRTT) performance probe trials with 1.5-, 1.0-, 0.8- and 0.6-second stimulus durations. After completion of the training, mice were randomized to either sham or rmTBI injury group. The probe trials were carried out at 2 w (**d**), 6 w (**e**) and 10 w post rmTBI (**f**), respectively. The response accuracy was compared between sham and rmTBI groups. n=10 for rmTBI; n=8 for shams. * denotes statistically significant difference from shams, p<0.05; F(3,45 = 2.861; 2-way ANOVA)

Behavioral flexibility, the capacity to change a behavior based on changes of rules and contingencies, was investigated in injured mice using the PVD-R task. At 2 weeks post-rmTBI or sham procedure the mice were baselined (B3 in Fig. 6b) and then the stimulus-reward contingencies were reversed and performance tested one session a day for 10 days (Fig. 6a–c). At 6 w post-injury the mice were baselined again and then the stimulus rewards switched back to the original reward profile and the mice tested again. As shown in Fig. 6 the rmTBI mice performed as well as the shams on the post-rmTBI baselining (B3) suggesting that rmTBI did not affect the ability to perform a previously learned PVD task. The reversal PVD performance at 2 weeks and 6 weeks also did not differ between rmTBI mice and shams, suggesting that rmTBI does not affect behavioral flexibility.

In contrast, we found pronounced deficits in attention. At 2 w post-injury there were no differences in performance of the 5-CSRTT between rmTBI and sham mice (Fig. 6d). However, at 6 w post-injury the rmTBI mice show reduced accuracy in performance of this task compared to shams at stimulus durations of 0.8 and 0.6 s (Fig. 6e). This reduction in performance accuracy in the rmTBI mice was not significant at 10 w (albeit rmTBI mice tended to perform slightly worse than controls) (Fig. 6f). There was no difference between shams and rmTBI mice in their premature response rates (total number of responses during the 5 s inter-trial interval per trial; F(93,45) = 0.508, *p* = 0.68) a measure of impulsivity or in their rates of preservative responses (total number of nose pokes before reward collection per trial; F(3,48) = 1.586, *p* = 0.205). The percent of omissions, reward collection latencies and correct touch latencies were also not significantly different between shams and rmTBI mice (all F < 1, *p* > 0.1). Thus, after five mTBIs at one day intervals mice demonstrate attentional deficits at 6 w post-injury that improved somewhat by 10 w post-injury.

### Repeated mTBI leads to deficits in spatial memory

**Fig. 7:**
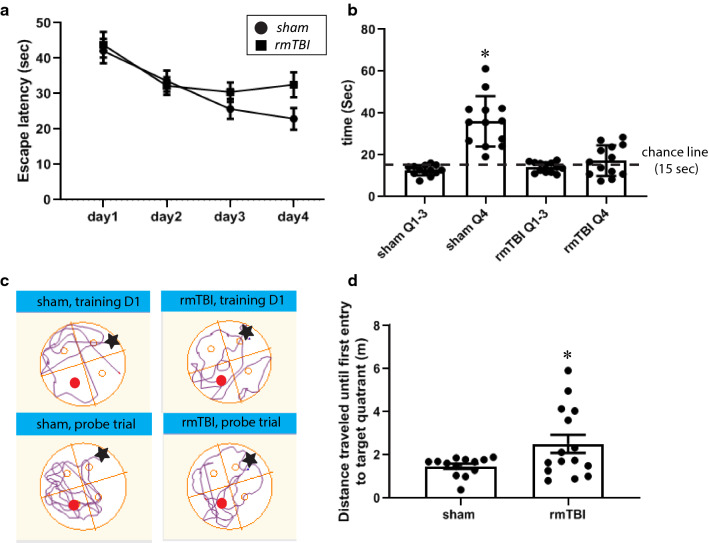
Morris Water maze deficits in rm-TBI mice. At 16-week post rmTBI, all the mice were trained for 4 consecutive days (4 sessions per day; platform in Quadrant 4) and performed probe test on day 5. **a** There was no difference between shams and rmTBI mice in escape latency during the training phase of the test (2-way ANOVA for A (F(3,108) = 1.127, *p* = 0.34). **b** Sham but not rmTBI mice spent more time in the target quadrant (Q4) compared to the average time spent in the non-target quadrants (Q1–3) during the probe trial (One-way ANOVA for b (F(3,48) = 29.13, *p* < 0.001). The time that a mouse might spend in any one quadrant by chance (15 s out of the 60 allotted for the trial) is shown by the dashed line. **c** Representative movement traced at day 1 training and probe trial from sham mouse and rmTBI mouse; red dot indicated the location of the hidden platform (in Q4); black star indicated the location where the mouse was released into to the pool (in Q2). **d** RmTBI mice traveled a greater distance than sham mice until their first entry into the target quadrant during the probe trial (Student *t*-test). Asterisk denotes statistically significant difference compared to shams *p* < 0.05

We and others have shown defects in spatial cognition and memory in mice in various models of mTBI [12, 17, 64, 96, 97]. We tested spatial cognition in the rmTBI mice at 16 w post-injury using the Morris Water Maze. Both sham and rmTBI mice showed decreased latencies for finding the hidden platform over the 4 days of training, indicating that spatial learning was not significantly impaired by the rmTBI (Fig. 7a). On the probe trial held on day 5, whereas sham mice spent significantly more time in the target quadrant than any of the other quadrants, mice in the mTBI group spent the same amount of time in the target quadrant as the average time spent in the other 3 quadrants (Fig. 7b, c). This was reflected in rmTBI mice traveling significantly further before their first entry into the target quadrant during the probe trial (Fig. 7d). The mean swim speeds of shams (0.23 ± 0.028 m/s) and rmTBI mice (0.21 ± 0.027 m/s) were not significantly different from each other. This suggests that the rmTBI mice had deficits in spatial memory.

### rmTBI leads to increased levels of pathological phospho-tau

**Fig. 8:**
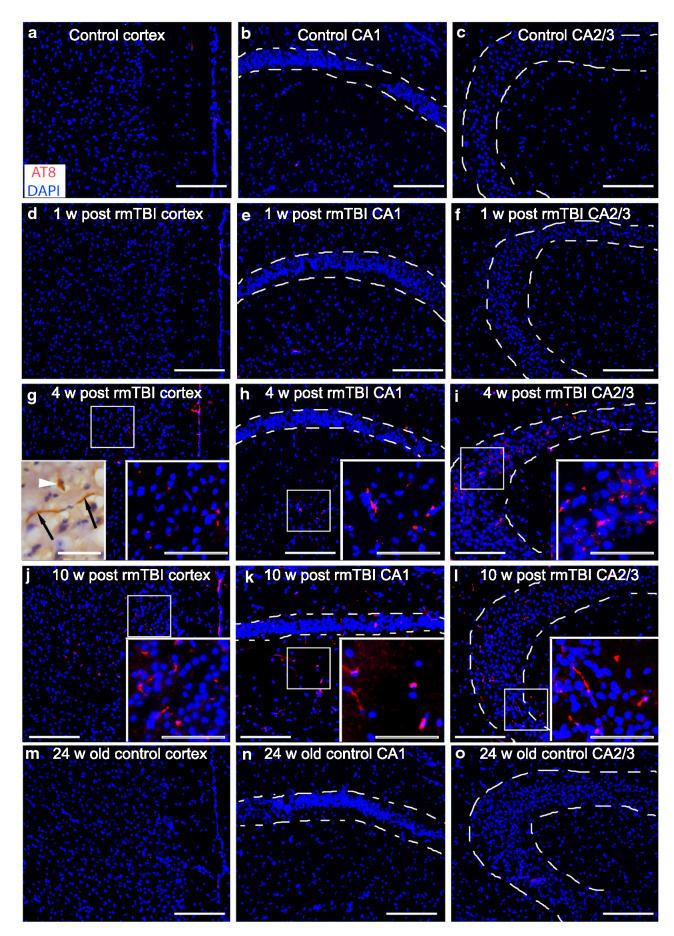
Pathological phospho-tau at 4 and 10 w post-rmTBI. Representative photomicrographs of AT8 immunostaining for pathologically phosphorylated tau (red) showed that while brain sections from controls (**a**–**c**) and rmTBI mice 1 week post-injury (**d**–**f**) did not demonstrate any detectable AT8 immunostaining, the cortex, CA1 (outlined by dashed lines in **b**, **e**, **h**, **k** and **n**) and CA2/CA3 regions (outlined by dashed lines in **c**, **f**, **i**, **l**, and **o**) from mice 4 w and 10 w post-rmTBI showed positive AT8 immunostaining (**g**–**l**). To better analyze the morphology of the stained stuctures select slides from the PFC were also stained using a DAB reaction. High magnification of these stained slides (inset in **g**) show positive perinuclear (white arrowhead) and fiber (black arrows) AT8 immunostaining. Sections from the cortex and hippocampus of control mice at 24 w of age (age-matched to the 10 week post-injury group) were negative for AT8 immunostaining suggesting that the AT8 immunostaining in the 4 and 10 w post-rmTBI groups was not due to their increased age (**m**–**o**). Scale bar panels A-E = 50 μm, scale bar for all insets = 25 μm

Tau is a microtubule associated protein that is important for stabilizing tubulin polymers that are essential for neuronal structure, axonal transport and plasticity [52]. Tau becomes pathologically phosphorylated after TBI [42] and is considered a neuropathological hallmark, if not the cause, in a range of neurodegenerative diseases [89]. Given the diffuse axonal injury revealed by silver staining and APP immunoreactivity in the brains of the rmTBI mice, we sought to determine whether Tau was hyperphosphorylated in the brains of the rmTBI mice by immunohistochemistry using the AT8 antibody that recognizes hyperphosphorylated Tau [29, 82]. AT8 immunoreactivity was undetectable in sections from control brains (12–14 week old mice) and sections from rmTBI mice at 1 week post-injury (Fig. 8a, b). However AT8 immunoreactivity was clearly evident in sections of rmTBI cortex and hippocampus at 4 and 10 w post-injury (Fig. 8c, d). Brain sections from control 24 week old mice (the age of the rmTBI mice 10 w after their injury was 22–24 weeks old) failed to show AT8 immunoreactivity (Fig. 8e) suggesting that the AT8 immunoreactivity in the rmTBI mice was not due to aging but to the rmTBIs they experienced.

### rmTBI leads to significant changes in blood metabolites

**Fig. 9:**
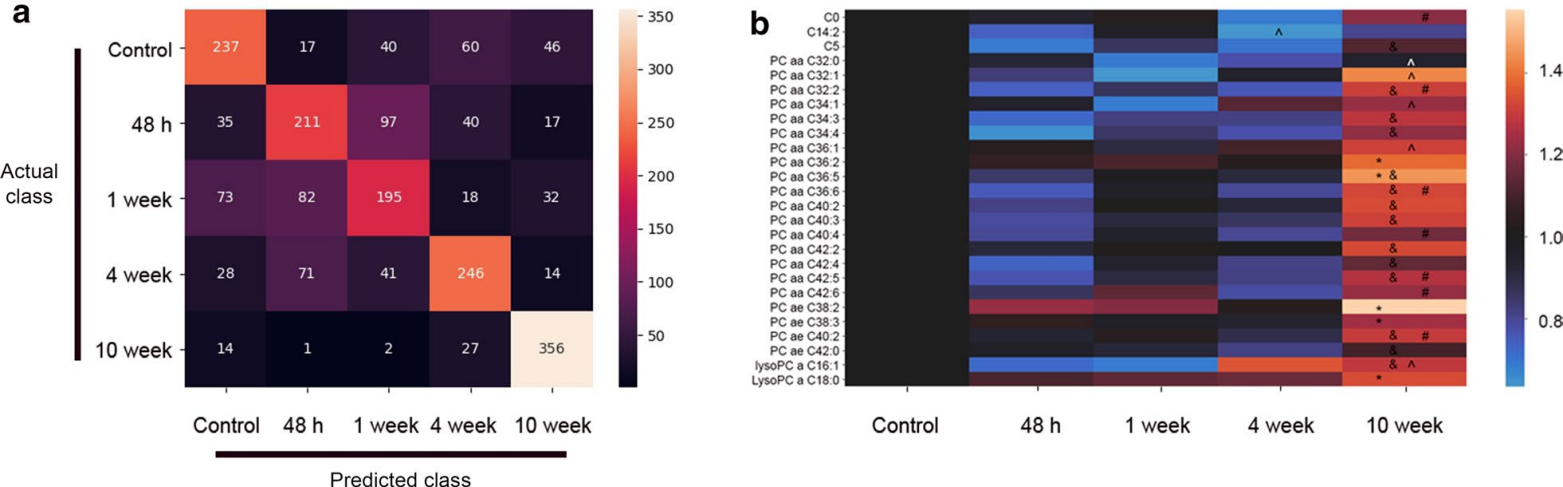
Long-term metabolomic abnormalities after rmTBI. **a** Confusion matrix using a random forest classifier demonstrates the accuracy with which metabolomics can be used to classify mice as either control, 48 h, 1 week, 4 w or 10 w post-rmTBI. The greatest accuracy was achieved for metabolomic profiles from animals 10 post-rmTBI (89%). A Mann–Whitney U Test was performed looking for statistically significant differences between every pair of time points for each metabolite. A Bonferroni correction was then used to correct the family-wise error rate. After the correction, there were 32 points of statistical significance spread across 26 metabolites. **b** A heat map displaying the levels of these metabolites relative to control levels (black) are shown. Almost all of the metabolites (23/26) are phosphatidylcholines or lyso-phosphatidylcholines and show similar patterns of expression: low (blue hues) early after rm-TBI and significantly higher (reddish hues) at 10 w after rmTBI

It has previously been shown that plasma metabolomics analyses coupled with machine learning can be used to classify boys playing Bantam hockey into concussed and non-concussed groups with an accuracy of 92% [21]. To determine whether applying supervised machine learning to the mouse metabolomics data could similarly be used to classify mice into one of the 5 possible categories (control, 48 h, 1, 4, or 10 w post-concussion), we collected blood samples from all groups of mice and subjected them to a metabolomics screen using the Biocrates kit. Blood metabolite levels were scaled using the StandardScaler package from Sklearn. A random forest classifier using the scaled blood levels of all 144 metabolites, 30 trees and a depth of 150 was used to classify the metabolomic signatures from each mouse sample as either control, 48 h, 1, 4, or 10 w post-injury. The data was randomly shuffled and split into training and testing data using 8 samples per time point as training data and the remaining 4 were used for the testing (n = 12 per group). This cross-validation (shuffling, splitting, training, and testing) was done 20 times using 20 random states for the data. The results were compiled into a confusion matrix (Fig. 9a). The accuracy for classifying blood samples from mice into the proper category ranged from 49% for samples taken from mice 1 week post-injury to 89% for blood samples from mice 10 w post-injury which are considerably better than the accuracy level of 20% expected when randomly guessing classifications across 5 groups. To determine which metabolites might show statistically significant differences between groups we performed a Mann–Whitney U Test on the levels of all 144 metabolites. After a Bonferroni correction for multiple comparisons 32 points of statistical significance were found for 26 of the metabolites. A heat map depicting the changes in expression levels of these metabolites (Fig. 9b) demonstrates that almost all of the metabolites that were found to be statistically different at one time point between groups were either phosphatidylcholines or lyso-phosphatidylcholines (23/26). These are the same class of phosphoglycerolipids that we previously found to differentiate between concussed and non-concussed Bantam hockey players [21]. These metabolites all showed a remarkably similar pattern of change after injury with levels initially being somewhat reduced and then being significantly elevated at 10 w post-injury.

### rmTBI leads to long-term white matter pathology

**Fig. 10:**
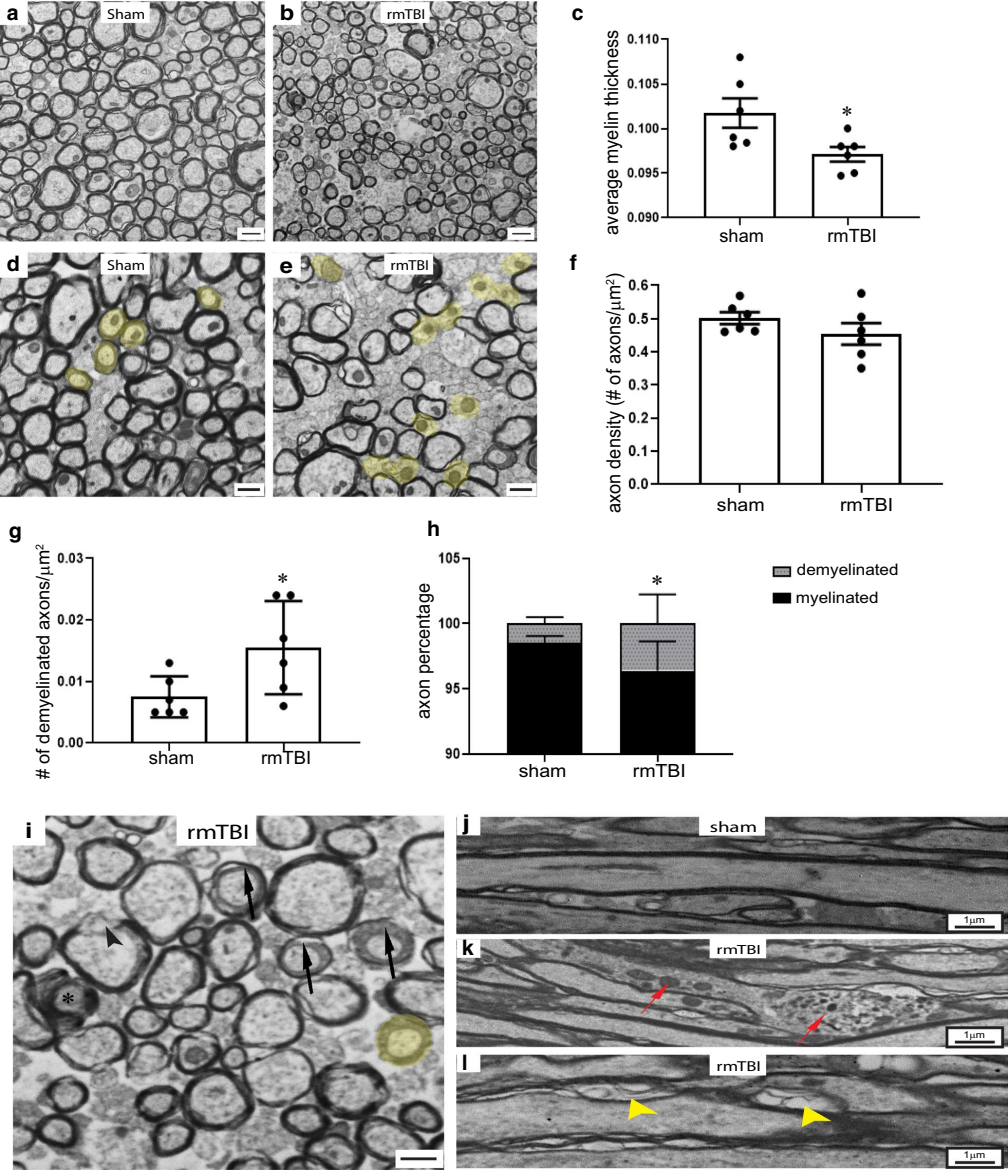
Long-term white matter pathology shown in EM. Saggital sections through corpus callosum were examined by electron microscopy at 20 w post-injury. **a** In sham animals, axons were tightly compact, most of which were myelinated. **b** In rmTBI mice axons were less compact, with dispersed degenerating axons and demyelinated axons. **c** The average myelin thickness was significantly reduced in the rmTBI mice. **d**, **e** Demyelinated axons (diameter > 0.3 mm with no detectable compact myelin) were identified in sham and rmTBI sections (light yellow). **f** The total axon density in sham and rmTBI mice were not different from each other. However, the density and percentage of demyelinated axons was significantly higher in the rmTBI samples (**g**, **h**). Asterisk denotes statistically signficant different from shams, Student *t*-test; *p* < 0.05. Representative electron micrographs of corpus callosum exhibited various abnormalities in **i**, decompaction (arrowhead); dystrophic axon (asterisk); demyelinated axon (light yellow) and separation of myelin from axon (arrow). Coronal sections through corpus callosum demonstrated uniform cytoskeletal structure in sham axons (**j**); accumulation of vesicles in degenerating axons (**k**, red arrows) and myelin decompaction (**l**, yellow arrow heads) were found after rmTBI. Scale bar = 1 μm in **a**, **b**; scale bar = 300 nm in **d**, **e**, **i**

Acutely after rmTBI our analyses demonstrated acute DAI accompanied by a short-lived inflammatory response. Behavioral testing, on the other hand showed deficits at 6 w (5-CSRTT) and at 16 w (spatial memory) post-injury. To investigate possible long-term pathological changes in the mTBI mice at 20 w post-injury we carried out ultrastructural analyses on a subset of the sham and rmTBI mice that had undergone the 5-CSRTT. Using Image Pro software average myelin thickness of axons that fell on the lines of a grid superimposed on each electron micrograph were measured (Fig. 10a, b, d, e) taken from the corpora callosa of shams and rmTBI (n = 6 per group) mice. The rmTBI mice had a significant reduction in their average myelin thickness (Fig. 10c). Inspection of the electron micrographs suggested that there was an increase in the number of demyelinated fibers in the corpora callosa of rmTBI mice. We therefore did a manual count of demyelinated and myelinated fibers in the electron micrographs and found that while the number of axons per unit area in the corpora callosa did not differ between shams and rmTBI mice, the number and percentage of demyelinated axons was significantly greater in rmTBI mice (Fig. 10f–h). Other white matter ultrastructural abnormalities were also frequently found in sections from rmTBI mice including myelin decompaction, increases in adaxonal spaces and axonal degeneration (Fig. 10i–l).

## Discussion

We set out to characterize short term and long term pathological and behavioural consequences of rmTBI in mice using a severity of injury predicted to be analogous to subconcussive injuries in humans. The mTBI used in this study produced modest linear and angular accelerations of 14.0 ± 2.2 g and 36.6 ± 24.5 krad/s^2^, respectively. By comparison, the CHIMERA model of mouse TBI produces linear accelerations of 524 g and angular accelerations of 149 krad/s^2^ [75]. Following an equal-stress equal-velocity relationship with a scaling factor of *λ* = (mass of human brain/mass of mouse brain)^1/3^ = 13.8 [79], we estimate that the equivalent human injuries would have peak linear accelerations of 1.0 ± 0.2 g, and peak angular accelerations of 0.2 ± 0.1 krad/s^2^. These linear and angular accelerations are approximately 40-fold less than the average linear acceleration (greater than 40 g) and peak angular accelerations (5.022 krad/s^2^) reported in human sports-related concussion [88]. In a pilot study using this model of mTBI, we found that a single mTBI failed to show any diffuse axonal injury in the corpus callosum of injured mice by silver staining 1 week after the mTBI. Thus, we judged our model of rmTBI to be exceedingly mild and perhaps representative of subconcussive injuries in people. Thus, with the intention of studying a mouse model of repetitive subconcussive head injuries, we subjected mice to 5 mTBIs (one a day for 5 days), predicting that it would produce only acute and subtle pathological and behavioral defects.

In keeping with that prediction many acute pathological changes were observed to be transient in our rmTBI mice. Silver staining and APP immunoreactivity of sections from the rmTBI mice showed that the injured mice had indeed the signs of DAI but that those signs of DAI tended to improve with time. Thus, the silver staining in the corpus callosum of injured mice was most intense at 1 week post-injury but declined by 10 w post-injury. Likewise APP immuno-staining was most obvious at 48 h and 1 w post-injury but largely absent by 4 w post-injury. The observation of intense silver staining after rmTBI but not after a single mTBI, suggests that, at least when spaced at one day intervals, multiple mTBIs have an accumulative effect. The DTI studies validate the transient nature of the DAI showing increased FA in the hippocampus and the corpus callosum acutely after injury, that return to baseline levels by 4 w post-injury.

Much like the evidence of DAI, the inflammatory response in the rmTBI mice was transient. Western blot analyses showed that GFAP and Iba1 expression, reflective of astrocyte and microglial activation respectively, were elevated twofold above sham levels at 8 h after the last of the 5 mTBIs but returned to baseline levels by 48 h post-injury. Proinflammatory cytokine expression (TNFα and IL-6) likewise only showed acute elevations returning to baseline levels by 1 w post-injury. The transient nature of this inflammatory response contrasts with the more persistent inflammatory response observed in more severe single [75] and repetitive mTBI models [17, 18] and further attests to the mildness of the present injury model.

The DAI evident by the silver staining and in particular the observation of silver-stained “corkscrew” axons in some of the corpus callosum sections led us to predict that the injury may have caused a disconnection between axon terminals and their synaptic targets. In support of this prediction, synaptophysin levels decreased acutely after rmTBI and this change was still apparent at 20 w post-injury. The acute elevation in PSD95 levels and reduction in Gephyrin levels may be taken to indicate alterations in the ratio of excitatory and inhibitory synapses as has also been suggested to occur after TBI [7, 11, 15, 19, 110]. We cannot rule out that these changes in synaptophysin, PSD95 and Gephyrin levels are due to changes in protein expression levels and not in synapse number however, others have shown that nerve terminals can be quantified by the levels of these nerve terminal markers [108], and these results certainly suggest an injury-induced increase in synaptic plasticity and remodeling. In particular, these data suggest that after rmTBI there is a temporary preferential loss of inhibitory synapses and a more robust regeneration of excitatory synapses, resulting in a temporary increase in the ratio of excitatory:inhibitory activity. A change in the balance of cortical excitatory and inhibitory activity after injury has been described by others [19] with many showing a specific loss of inhibitory synaptic activity [7, 11, 15, 110]. The PSD95 and Gephyrin levels return to baseline by 1 w after rmTBI and might thereby restore the balance of excitatory and inhibitory synaptic activity. However it would also seem possible that the injury-induced remodeling may not have returned individual circuits to their pre-injury states. Furthermore, the long-term reductions in synaptophysin levels suggest an overall loss of synapses due to rmTBI that are not fully compensated for even by 20 w post-injury. Alterations and/or deficiencies in the remodeling of individual circuits could explain the long-lived changes in cognition and behavior observed in this experimental paradigm.

In the current study, region of interest analyses following in-vivo DTI revealed transient increases in FA and decreases in RD in a volume encompassing hippocampus and the white matter of the corpus callosum. Increased FA in the corpus callosum and the hippocampus of rats following a weight drop TBI has similarly been reported [49]. Moreover, increased FA in the corpus callosum has also been reported in human mTBI [8, 37, 65, 80]. These changes have generally been associated with neuroinflammation or remyelination [57, 90]. In the present study the increased FA may be attributed to the transient neuroinflammation identified by the Western blot and immunohistochemical analyses. The increased FA that we observed in the rmTBI mice might also be attributed, to the acute astrogliosis evident by increased GFAP levels at 8 h post-injury as others have suggested that astrocyte proliferation is the main cause of increased FA after TBI [56].

While the DAI and inflammation induced by the rmTBI injury were either resolved or greatly improved by 4 w after injury, behavioral testing and MRS suggested prolonged consequences of the injury. The 5-CSRTT demonstrated attention deficits in the rmTBI mice at 6 w post-injury that improved by 10 w post-injury. While we did not observe any differences between shams and rmTBI mice on the PVD-R, others have reported a defect in rmTBI mice on this task [83]. In their study Pinkowski et al. first exposed the mice to rmTBI (4 mTBIs, one a day for 4 days) and then two days later began the PVD training. The rmTBI mice had significant difficulties learning this task using this paradigm [83]. In the present study we first trained the mice on the PVD task until they reached a criterion of 80% correct choices for 2 days in a row before being randomized to either the sham or rmTBI groups. Thus, while the work of Pinkowski et al. tested whether learning a PVD task was impaired by rmTBI, our paradigm tested whether rmTBI affected the ability to perform a PVD task already learnt and whether rmTBI affected cognitive flexibility when the rules were changed in an already learnt PVD task. Our findings clearly show that rmTBI does not interfere with performance on an already learnt PVD task nor does it interfere with cognitive flexibility. The finding of attention deficits in the rmTBI mice but not of behavioral flexibility suggests that, as in the case of human concussion, the rmTBI have rather subtle, high-level cognitive deficits that may exist in one cognitive domain but not in others. The specificity of the cognitive deficits in the rmTBI mice might be due to the location of the injury or its severity but will likely be better understood in the context of the pathology in the specific circuits that underlie these different cognitive functions. As the PFC has been shown to be critical to performance on tasks of attention [23, 58] and our analyses demonstrates that the rmTBI triggered synaptic remodeling specifically in the PFC, we suggest that some of this remodeling may have impaired attention in these mice.

Late-developing cognitive defects in the rmTBI mice was also revealed by the testing in the Morris water maze at 16 w post-injury. In this test of spatial memory, experiments in rmTBI mice revealed that they were able to learn the location of the hidden platform as well as the sham control mice during training but that they were deficient in remembering the task the next day during the probe trial. Others have also shown long-term deficits in spatial cognition using the Morris water maze in a more severe model of mTBI [17].

Prolonged changes were also observed in the in-vivo MRS measures. For example, reduced lactate was observed at 10 w compared to 48 h post-injury. Lactate levels have been shown to increase in TBI [54, 99], due to the presence of hypoxic/ischemic conditions. It has also been shown that inhibition of lactate production in the brain of mice can lead to impaired spatial learning and memory using the Morris Water Maze [36]. Thus, the lower lactate levels in the hippocampus of rmTBI mice observed by MRS at 10 w post-injury may explain their poor spatial memory performance. Increased Glu/Cr and Gln/Cr ratios were also observed over the 10-week period post-injury. Metabolite ratios may be highly sensitive to subtle changes when metabolite levels move in opposite directions, however interpretation of these changes can be challenging. The significant increase in Gln/Cr is likely due to a small increase in glutamine, and a concomitant reduction in creatine. Moreover, this change in Gln/Cr level is consistent with that observed in a previous study of varsity rugby athletes in comparison to non-contact athletes [92]. We speculate that the five mTBIs paradigm used in the present study may simulate the impact conditions that contact athletes experience over a season of play, eventually causing elevated glutamine levels.

The observation that rmTBI mice showed cognitive defects at a time after injury when markers of DAI and inflammation were declining or already at baseline levels suggested that the initial injury may have triggered pathological processes that worsen with time. The levels of pathological phospho-tau increased with time in rmTBI; AT8-immunostaining for phosphor-tau was not detectable in controls or in rmTBI mice at 1 week post-injury but increased in rmTBI brain sections taken at 4 and 10 w post-injury. Importantly, AT8 phospho-tau immunostaining was not detectable in mice age-matched to the 10 week post-rmTBI mice. This persistent increase in pathological phospho-tau has been seen in other models of rmTBI in mice [89]. The finding that tau phosphorylation increased with time in the present model of rmTBI is reminiscent of human concussion in which phospho-tau is considered the hallmark of chronic traumatic encephalopathy (CTE), a form of dementia associated with a history of repetitive mild TBI [34, 68, 69].

In addition to the pathological phospho-tau found at chronic time points after injury, we also found evidence of white matter damage at 20 w post-rmTBI. The ultrastructural changes in the white matter included a decrease in myelin thickness and a decrease in the percentage of axons that were myelinated despite the fact that axon number were not different from shams. In addition, we observed that in the corpus callosum of rmTBI mice the myelin was decompacted, frayed and had, in many cases, pulled away from the axon creating an increased adaxonal space. There were also many instances of axonal degeneration as evidenced by accumulations of vesicles. These types of white matter and axonal pathologies at the ultrastructural levels have been shown in concussed mice at earlier time points post-injury [71] and demonstrates that repeated mTBI in mice can result in white matter pathologies that have not resolved even by 20 w post-injury.

The cognitive testing, MRS, Western blot analyses for synaptic markers, ultrastructural analyses and phospho-tau immunohistochemistry collectively suggest that even exceptionally mild repeated TBIs can result in long-term changes in the brain that worsen with time. The metabolomics data supports this view. Using a random forest classifier we were able to show that metabolic signatures in the blood could be used to classify mice as either controls or 48 h or 1 or 4 or 10 w post-injury with accuracies that ranged from 49 to 89%. These classification accuracies are considerably better than chance (20%). Furthermore we found that phosphatidylcholines and lyso-phosphatidylcholines made up 23 of the 26 metabolites that were statistically different at at least one time point studied. Phosphatidylcholines have been shown to be important in the classification of human concussion [21, 26] and their co-ordinate patterns of expression in the rmTBI mice suggests that this family of metabolites are co-regulated after injury. The fact that all of these metabolites were statistically significantly up-regulated at 10 w post-injury reinforces the idea that repeated mTBI leads to long-term changes that do not resolve to baseline but perhaps either continue to evolve over time or evolve until establishing a new post-mTBI baseline that is beyond the time points studied here.

There are many parallels between the cognitive and pathological changes reported in human concussion and the cognitive and pathological changes we have described in the present mouse model of rmTBI. For example, attention and memory deficits are well-known challenges for concussed patients [40, 73, 98] and are deficient in the rmTBI mice. DTI in the rmTBI mice shows increases in fractional anisotropy (FA), an indication of white matter pathology, at 48 h (in the corpus callosum and hippocampus) and at 1 w post-concussion (in the hippocampus). These results mirror those in the majority of DTI studies in concussed athletes that also show early increases in FA [16, 67, 109]. The increased FA and reduced RD in the current study are also consistent with our own previous findings in *non-concussed* contact sport athletes [92]. Specifically, we observed increased FA and reduced RD within the white matter of the MRS voxel in female rugby players after a season of contact play. Interestingly, a correlation was observed between the change in Gln/Cr and the change in FA and RD over the sports season. We observed a similar correlation in the current study between glutamine and RD at 10 w. The neuropathological studies in the rmTBI mice also suggest parallels with the neuropathology of human concussion. For example silver and anti-APP staining of rmTBI brain sections shows that DAI can be detected as early as 48 h post-rmTBI (the earliest time point analyzed) and persists for weeks. APP accumulations after TBI is a well-known diagnostic feature in human DAI [101, 106]. Finally the pathological phospho-tau accumulations in the rmTBI mice is mirrored in humans by the observation that after an acute TBI in humans there is an increase in tau levels and tau phosphorylation [42]. In long-term survivors of TBI, 34% of people examined demonstrated tau pathology compared to 9% in age-matched controls [44]. Furthermore, aggregations of phospho-tau is considered the hallmark of chronic traumatic encephalopathy (CTE), a form of dementia associated with a history of repetitive mild TBI (including those caused by blast injury) [34, 68, 69].

## Conclusions

The mouse model of rmTBI described in this paper is exceedingly mild as judged by the kinematic measurements made at the time of injury and by the transient nature of the inflammation that the injury triggered. At the same time our rmTBI model led to cognitive deficits, pathological changes in the white matter, increased pathological phospho-tau levels and changes in metabolite levels that lasted for months after the injury. It is possible that rmTBI as used here was not as mild as the kinematic measurements suggest and that differences in the ratios of white matter to gray matter or in the geometries of the brain and skull or in the presence of cortical gyri between mice and humans confound our ability to scale TBIs appropriately between humans and mice [31, 79]. This argument would suggest that we exercise great caution in scaling human TBI down to the mouse based on kinematic measurements alone and that other biomarkers of neuropathology such as cognitive function, and measurements of white and gray injury need to be further refined and more heavily weighted. However, the modest and short-lived inflammatory response triggered by the rmTBI model described here favors the explanation that the rmTBIs were indeed mild but that nonetheless they triggered pathophysiological changes that evolved with time and lasted months. When extrapolated to the human case, these findings suggest that should a person experience rmTBIs, whether or not they be diagnosed with concussion, that the repetitive nature of the injury may trigger pathological processes with long-term pathological and cognitive consequences. This prediction is supported by our own studies showing long-term changes in fMRI, DTI and MRS in non-concussed women rugby players [62, 63, 92]. It is also supported by the findings of cognitive impairments in athletes that are supposedly concussion-free [50, 66, 104]. This work brings into focus that exceedingly mTBIs may escape diagnosis but still trigger pathological consequences that may not be manifest until a much later time. Thus, the mouse model described may be useful in studying the effects of rmTBIs that may occur most commonly—the undiagnosed mTBIs that accompany regular contact sport play.

## Supplementary Information

**Additional file 1. Fig. S1**: Evaluating the inflammatory markers in brain samples from Naïve mice and shams sacrificed at 8 and 48 h after the last sham procedure. Western blot and subsequent densitometric analyses show the levels of GFAP (**a**), Iba1 (**b**), TNFα (**c**) and IL-6 (**d**) levels in brain samples from the prefrontal cortex of naïve mice and mice sacrificed 8 h and 48 h after their last sham procedure. β-actin levels were used as a loading control. None of the protein levels were found to be statistically different between any of the groups, n = 4–5 per group, *p* < 0.05; one-way ANOVA).

## Data Availability

The touchscreen datasets generated and analyzed during the current study will be available in the MouseBytes repository (https://mousebytes.ca/home) [9]. The other datasets generated and analyzed during the current study will be deposited in Figshare (https://figshare.com/account/home).

## Abbreviations

5-CSRTT: 5-Choice serial reaction time test
APP: Amyloid precursor protein
Cr: Creatine
CTE: Chronic traumatic encephalopathy
DAI: Diffuse axonal injury
DTI: Diffusion tensor imaging
FA: Fractional anisotropy
fMRI: Functional magnetic imaging
GCS: Glasgow coma scale
Gln: Glutamine
Glu: Glutamate
h: Hours
IL-6: Interleukin-6
mTBI: Mild traumatic brain injury
MRS: Magnetic resonance spectroscopy
NAA: N-acetyl aspartic acid
PFC: Prefrontal cortex
PVD-R: Paired visual discrimination task with reversal
RD: Radial diffusivity
rmTBI: Repetitive mild traumatic brain injury
ROI: Region of interest
TBI: Traumatic brain injury
TNFα: Tumor necrosis factor alpha
w: Weeks
