# Supplementary figures and images for: Repetitive mild traumatic brain injury in mice triggers a slowly developing cascade of long-term and persistent behavioral deficits and pathological changes
Source: Acta Neuropathol Commun. 2021 Apr 6;9:60. doi: 10.1186/s40478-021-01161-2 (PMC8025516; doi:10.1186/s40478-021-01161-2)

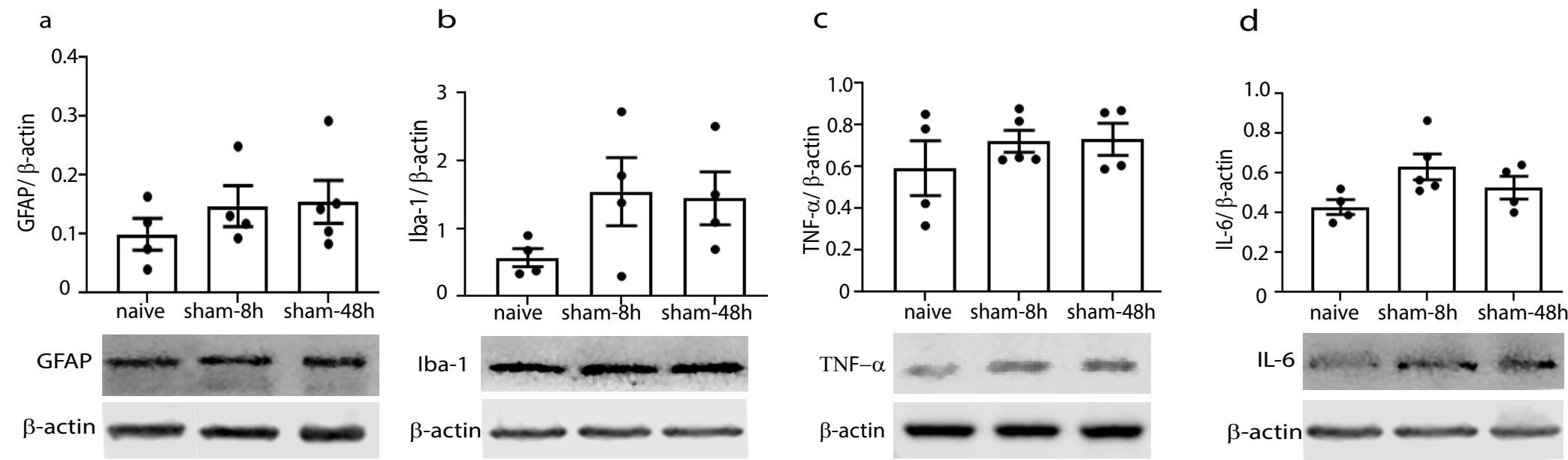

Supplement: Supplementary file 1 — Additional file 1. Fig. S1: Evaluating the inflammatory markers in brain samples from Naïve mice and shams sacrificed at 8 and 48 h after the last sham procedure. Western blot and subsequent densitometric analyses show the levels of GFAP (a), Iba1 (b), TNFα (c) and IL-6 (d) levels in brain samples from the prefrontal cortex of naïve mice and mice sacrificed 8 h and 48 h after their last sham procedure. β-actin levels were used as a loading control. None of the protein levels were found to be statistically different between any of the groups, n = 4–5 per group, p < 0.05; one-way ANOVA). [file 40478_2021_1161_MOESM1_ESM.pdf]
